# Physiological Essence of Magnesium in Plants and Its Widespread Deficiency in the Farming System of China

**DOI:** 10.3389/fpls.2022.802274

**Published:** 2022-04-25

**Authors:** Muhammad Ishfaq, Yongqi Wang, Minwen Yan, Zheng Wang, Liangquan Wu, Chunjian Li, Xuexian Li

**Affiliations:** ^1^Key Laboratory of Plant-Soil Interactions, College of Resources and Environmental Sciences, National Academy of Agriculture Green Development, Ministry of Education, China Agricultural University, Beijing, China; ^2^Shaanxi Forestry Bureau, Xi'an, China; ^3^International Magnesium Institute, Fujian Agriculture and Forestry University, Fuzhou, China

**Keywords:** magnesium deficiency, physiological functions, photosynthetic activity, crop yield and quality, Mg transporter family (MRS2/MGT), China's agricultural system

## Abstract

Magnesium (Mg) is an essential nutrient for a wide array of fundamental physiological and biochemical processes in plants. It largely involves chlorophyll synthesis, production, transportation, and utilization of photoassimilates, enzyme activation, and protein synthesis. As a multifaceted result of the introduction of high-yielding fertilizer-responsive cultivars, intensive cropping without replenishment of Mg, soil acidification, and exchangeable Mg (Ex-Mg) leaching, Mg has become a limiting nutrient for optimum crop production. However, little literature is available to better understand distinct responses of plants to Mg deficiency, the geographical distribution of soil Ex-Mg, and the degree of Mg deficiency. Here, we summarize the current state of knowledge of key plant responses to Mg availability and, as far as possible, highlight spatial Mg distribution and the magnitude of Mg deficiency in different cultivated regions of the world with a special focus on China. In particular, ~55% of arable lands in China are revealed Mg-deficient (< 120 mg kg^−1^ soil Ex-Mg), and Mg deficiency literally becomes increasingly severe from northern (227–488 mg kg^−1^) to southern (32–89 mg kg^−1^) China. Mg deficiency primarily traced back to higher depletion of soil Ex-Mg by fruits, vegetables, sugarcane, tubers, tea, and tobacco cultivated in tropical and subtropical climate zones. Further, each unit decline in soil pH from neutral reduced ~2-fold soil Ex-Mg. This article underscores the physiological importance of Mg, potential risks associated with Mg deficiency, and accordingly, to optimize fertilization strategies for higher crop productivity and better quality.

## Introduction

Since groundbreaking efforts in plant nutrition within the late nineteenth and twentieth centuries, complicated nutritional disorders and biological functions of most mineral nutrients are being systematically investigated. Research efforts have remained largely focused on crop yield- and quality-related, most frequently occurring limiting macro-elements such as nitrogen (N), phosphorus (P), and potassium (K). Nevertheless, the essentiality of other mineral nutrients, i.e., magnesium (Mg) has long been disregarded in terms of soil and plant tests and fertilization programs, and its deficiency is not regarded as a substantial concern in agriculture productivity. Consequently, Mg is called “a forgotten element in crop production” (Cakmak and Yazici, 2010). Indeed, Mg is an indispensable component in a large number of crucial physiological and biochemical processes throughout plant growth and development.

Mg is the fundamental component of chlorophyll (Chl) pigments in the light-capturing complex of chloroplasts and, hence, is involved in photosynthetic CO_2_ assimilation (Cakmak and Kirkby, 2008; Cakmak and Yazici, 2010; Gerendás and Führs, 2013). Nearly 15–35% of absorbed Mg by plants is fixed in Chl pigments, with the remaining (~65–85%) portion deposited in the vacuoles or utilized for protein synthesis and other related biological processes (Karley and White, 2009; Marschner, 2012). Mg also acts as a cofactor of numerous enzymes (more than 300) involved in Chl biosynthesis and photosynthetic CO_2_ fixation (Peng et al., 2015; Billard et al., 2016; Ma et al., 2016; Chen Z. C. et al., 2018). Various other enzymes such as ribulose-1,5-bisphosphate carboxylase/oxygenase (Rubisco), protein kinases, RNA polymerase, glutathione synthase, adenosine triphosphatases (ATPases), phosphatases, and carboxylases require Mg for activation (Maguire and Cowan, 2002; Shaul, 2002; Dann Iii et al., 2007; Marschner, 2012). Moreover, Mg participates in sucrose transport, energy metabolism, N utilization, pollen development and male fertility, stress tolerance, plant-microbe interactions, and other numerous biological processes (Li et al., 2008; Chen et al., 2009; Xu et al., 2015; Chen Z. C. et al., 2018; Li D. et al., 2020; Ishfaq et al., 2021; Tian et al., 2021).

Apart from plants, Mg is a key element for animal and human health. The suppression and rehabilitation of several human diseases, for instance, strokes, cardiovascular diseases, and diabetes involve the utilization of Mg (Al Alawi et al., 2018). Approximately two-thirds of adults in western countries do not consume satisfactory amounts of Mg, resulting in the nutritional disorder called “hypomagnesemia” that subsequently escalates to chronic diseases from its hidden deficiency (Hermans et al., 2013). The Mg-related nutritional disorders in humans are linked to declining Mg concentrations in the soil and food crops (Rosanoff, 2013; Rosanoff and Kumssa, 2020). Therefore, optimizing Mg nutrition is an imperative concern for food security and human health (Broadley and White, 2010).

The significance of Mg nutrition in crop production has long been overlooked and, consequently, its application seldom matches removal. Intensive Mg removal by high-yielding fertilizer-responsive cultivars and accelerated Mg leaching along with soil acidification continuously deplete indigenous exchangeable Mg reserves (Wang et al., 2020, 2022). Incidences of Mg deficiency (MGD) or approaching deficient levels is expanding in most production systems. As MGD is beginning to catch attention, still very limited literature is available to understand the biological functions and spatial distribution of Mg in distinct agroecosystems. Therefore, a better understanding of the physiological functions of Mg and the magnitude of MGD is vital to optimize future crop yield and quality.

Here, we compile research advances in Mg transport, physiology, and functions, and illustrate spatial patterning of MGD in diverse agricultural systems. In the first part of this study, plant morphological and related physiological responses to Mg availability are critically summarized. We also discussed the absorption and transport mechanism of Mg in view of the model plants *Arabidopsis* and rice. In the second part, spatial Mg distribution and magnitude of MGD in China's agricultural system are critically evaluated. There are also a few examples from other cultivated regions of the world, however, still scarce data is available to quantify them comprehensively. To our best knowledge, this is the first attempt of such kind that highlights geographical distribution and degree of MGD in distinct regions and even the provincial level in China. It can be beneficial to understand the overall biological functions of Mg, its nutritional status, and therefore, to optimize fertilization strategies as it highlights large spatial distribution variations. Thus, this review provides a unique reference for a wide range of scientific communities in plant sciences, and more critically draws attention toward potential threats associated with MGD for optimizing nutrient management to sustain agricultural productivity and food security.

## Threshold Levels of Magnesium in the Soil and Plant

Mg concentrations in the soil and plant are mediated by a wide range of intrinsic and extrinsic factors such as edaphic factors (soil type, pH, competing cations, moisture, aeration, etc.), environmental conditions (temperature, light, etc.), and anthropogenic influences (fertilization and other management practices). They also widely differ across plant species because of genotypic variations, heterogeneous growing conditions, growth stage, and age of analyzed tissues; hence, determining critical Mg levels is challenging (Gransee and Führs, 2013). For instance, different concentrations of Mg in leaves vary with temporal variations in Mg-deficient mulberry (Tewari et al., 2006), bean (Neuhaus et al., 2014), and barley (Tränkner et al., 2016) because Mg is a quite mobile element within the plant.

Concentrations of soil exchangeable Mg (Ex-Mg) fluctuate largely, and in most production systems more than 120 mg Ex-Mg kg^−1^ soil is generally considered sufficient for optimum crop yield (Metson and Gibson, 1977; Wang et al., 2020). In soil solution, values between 0.12 and 8.5 mM are categorized as satisfactory to support plant growth (Hariadi and Shabala, 2004; Karley and White, 2009; Marschner, 2012). Below this range, plant growth tends to be reduced, while overhead 8.5 mM can experience adverse influences (Hariadi and Shabala, 2004; Guo et al., 2016). In many plants, the optimum Mg concentration is 0.15–0.50% of leaf dry weight (Grzebisz, 2011; Marschner, 2012; Römheld, 2012); <0.1% is normally allied with chlorosis (Hermans and Verbruggen, 2005; Ding et al., 2006).

The leaf Mg concentration is found to be extremely species-dependent (Hauer-Jákli and Tränkner, 2019). The optimum Mg concentration in leaf dry weight falls in the range of 0.1 and 0.2% in rice, wheat, maize, barley, sorghum, and potato. While there is a usually higher demand of Mg for the dry matter formation in cotton, soybean, or peanut with the range between 0.2 and 0.3%, and even much higher 0.35% in the sunflower, alfalfa, and tomato. Based on dry matter formation, the optimum Mg concentration ranges 0.09–0.40% for woody plants, 0.07–0.21% for monocots, and 0.10–0.70% for dicots. Further, for optimum photosynthetic activity, woody species, monocots, and dicots require Mg concentrations between 0.10–0.5, 0.15–0.41, and 0.10–0.72%, respectively (Römheld, 2012; Hauer-Jákli and Tränkner, 2019).

The most obvious MGD symptoms generally appear on fully expanded mature (older) leaves and are critical tissues for MGD threshold setup. Nevertheless, interveinal chlorosis does not occur until a certain period of MGD latency and at that stage, it is literally difficult to attain full capacities of plant performance with Mg supplementation. Indeed, retarded root growth may serve as a reliable quicker indicator for MGD than chlorosis. Another much earlier and sensitive MGD indicator is elevated sugar concentrations in mature leaves which reduces photo-assimilate efflux and cumulatively reduces root growth (Hermans and Verbruggen, 2005; Koch et al., 2020). To better understand how plants respond to MGD, distinct plant responses regulated by Mg supply are summarized below.

## Responses of Plants to Magnesium Supply

**Figure 1:**
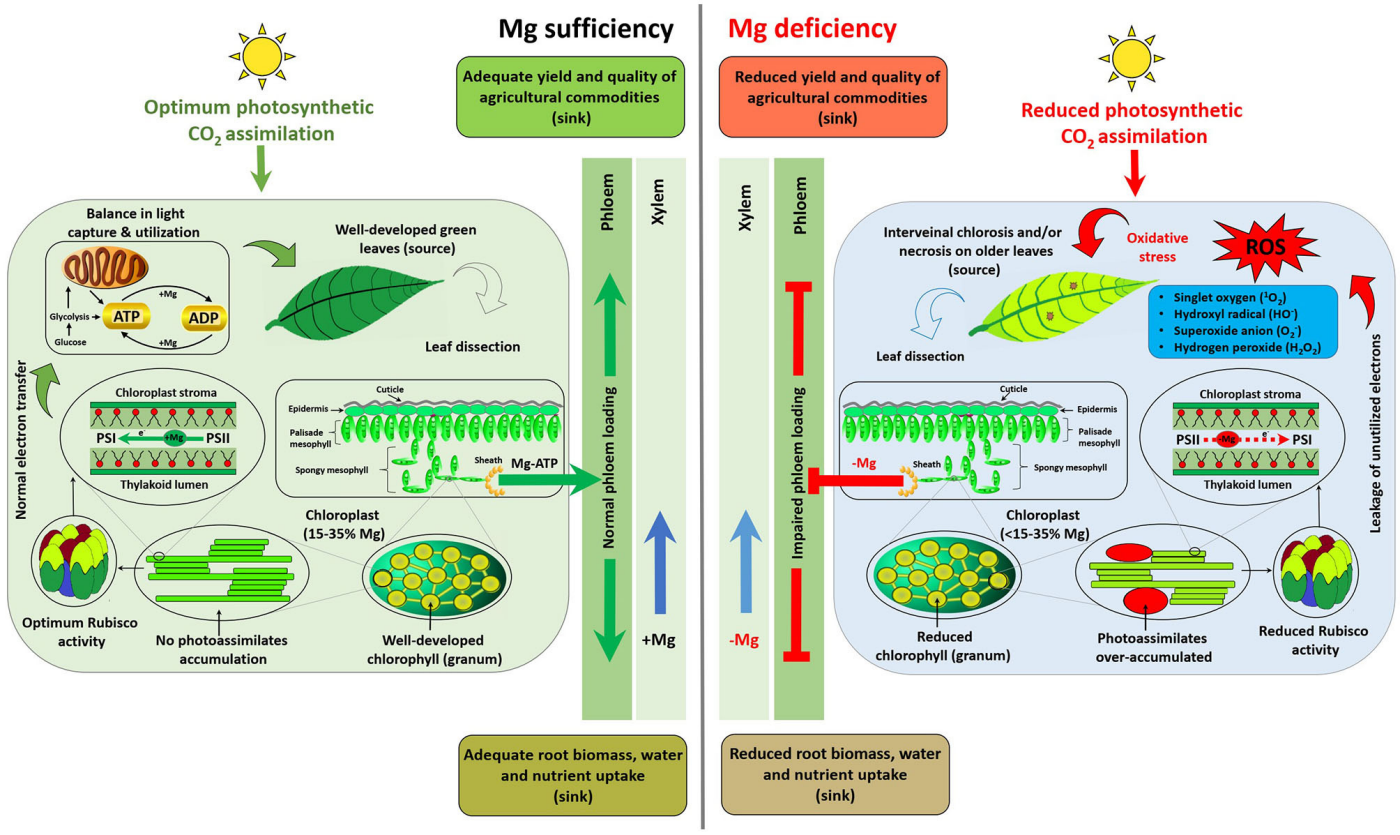
Scheme depicting distinct responses of plants to Mg availability. Plant response to MGD can be summarized as (1) sub-optimal photosynthetic CO_2_ assimilation; (2) impaired carbohydrate transportation from the source (fully expanded older leaves) to sinks (root, young growing points, or yield-forming organs); (3) photoassimilate (starch and sucrose) accumulation in source leaves; (4) reformed rounded and larger chloroplasts; (5) imbalanced light capture and utilization; (6) leakage of electrons from photosynthetic apparatus; (7) generation of reactive oxygen species (ROS); (8) oxidative stress; (9) degradation of chlorophyll (a & b) and reduced activities of Mg-depended enzymes, i.e., Rubisco; and (10) ultimately the appearance of interveinal chlorosis and/or necrotic spots on fully expanded (mature) leaves. Impaired phloem loading results in suppression of root growth, uptake of nutrients and water along with yield and quality traits of agricultural commodities.

Mg mediates various central physiological and biochemical processes, and in particular functions in the production, transportation, and utilization of photosynthates in plants. This section hierarchically summarizes comparative plant responses to Mg sufficiency and limitation (scheme depicted in Figure 1).

### Morphological Attributes Influenced by MGD

The well-recognized morphological disorders associated with MGD can be categorized as (1) shrinkage of the root system, (2) appearance of interveinal chlorosis, and/or necrosis typically on fully expanded (mature) leaves and (3) reduction in the yield and quality of agricultural commodities.

#### Shrinkage of the Root System

One of the distinct and earliest responses of plants to MGD is the noticeable shrinkage of the root system and the smaller root-to-shoot dry weight (DW) ratio, as a result of restricted photosynthate transportation from source leaves to sink organs (Cakmak et al., 1994a; Cakmak and Kirkby, 2008; Farhat et al., 2016; Ishfaq et al., 2021; Jia et al., 2021). MGD from the initial growth stage remarkably suppresses dry matter production by 34% in barley (Tränkner et al., 2016) and 43% in tomato seedlings (Ishfaq et al., 2021), which when sufficiently supplied, Mg improves root biomass by 77% compared to a smaller increase in shoot biomass (59%) (Hauer-Jákli and Tränkner, 2019). Disrupted root and shoot growth, and resulting decreases in the root/shoot ratio under MGD has been widely investigated (Cakmak et al., 1994a; Ding et al., 2006; Tewari et al., 2006; Mengutay et al., 2013; da Silva et al., 2017; Chen C. T. et al., 2018; Ishfaq et al., 2021; Xu et al., 2021). MGD hampers energy-requiring steps (i.e., Mg-ATP) for carbohydrate translocation toward the root (Section Impaired Carbohydrate Loading and Outflow for detailed description), leading to over-accumulation of carbohydrates in Mg-deficient leaves and substantial reduction of sucrose transport toward roots (Hermans et al., 2005; Koch et al., 2020). Further, down-tuning of auxin accumulation and signaling in Mg-deficient root preconditions a smaller root system (Ishfaq et al., 2021). A smaller root system under MGD diminishes the root surface area for resource forage in the soil, which further reduces uptake of other nutrients and increases the risk of multiple environmental stresses (Cakmak and Kirkby, 2008). However, Mg is a quite mobile element within the plant and older leaves (mostly in vacuoles) serve as a pool for actively growing points with higher requirements for Mg (Xu et al., 2021). Plants cultivated with sufficient Mg during the initial growth stages absorb and store sufficient Mg to endure upcoming MGD up to a certain extent, with minor changes in root/shoot ratios even if Mg supply is abruptly reduced (Hermans and Verbruggen, 2005; Niu et al., 2014; Tränkner et al., 2016; Liu et al., 2018). In addition, source-sink distance influences the translocation efficiency of carbohydrates and dry matter distribution, thus the ultimate root/shoot ratio may vary across plant species reliant upon the proximity among the source (leaves) and sink organs (Hermans et al., 2005).

#### Appearance of Leaf Interveinal Chlorosis

**Figure 2:**
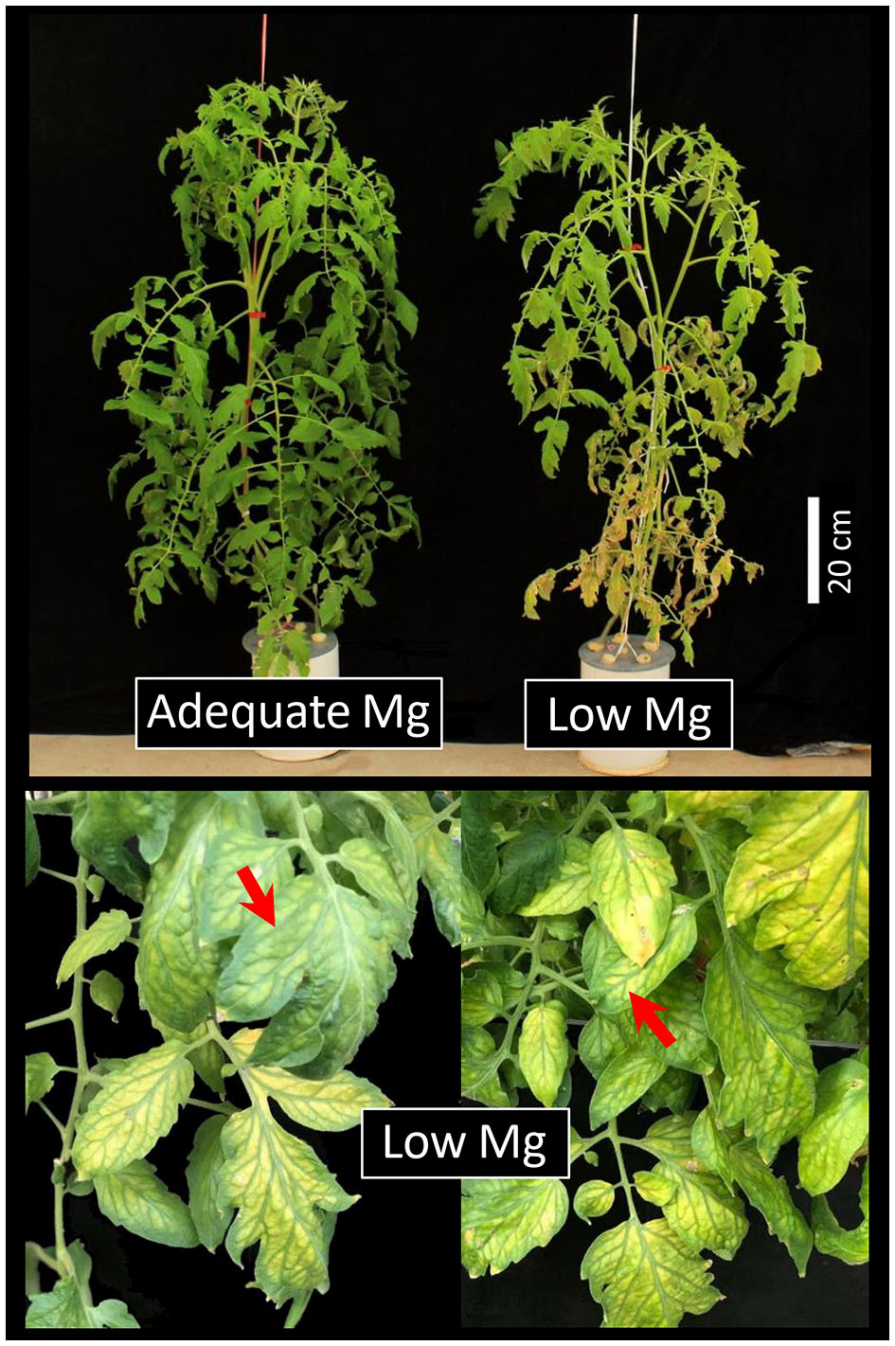
The visual Mg deficiency symptoms on tomato seedling. Under low Mg supply, interveinal chlorosis usually first appears on older, fully expanded (mature) leaves, and gradually encompasses younger leaves as Mg deficiency becomes more severe. Where adequate and low Mg were applied as 1.0 mM and 0.02 mM Mg, respectively, in the nutrient solution. Other details on plant growth have been described elsewhere (Ishfaq et al., 2021).

Mg acts as the fundamental atom of the Chl molecule; MGD leads to degradation of Chl pigments and subsequent interveinal chlorosis (Cakmak and Yazici, 2010; Hermans et al., 2013; Ye et al., 2019). Plants re-allocate Mg from older leaves toward actively growing younger leaves under MGD (White and Broadley, 2009; Taiz and Zeiger, 2010). Therefore, interveinal chlorosis usually first appears on older fully expanded (mature) leaves (Cakmak and Kirkby, 2008; White and Broadley, 2009; Gransee and Führs, 2013), and gradually encompasses young leaves as MGD becomes more severe (Billard et al., 2016; Li et al., 2017; Cai et al., 2019) (as shown in Figure 2).

Over-accumulation of photosynthates (starch and sucrose) in Mg-deficient leaves down-regulates the photosynthetic efficiency and utilization of fixed light energy (Sections Weakened Photosynthetic Activity and Impaired Carbohydrate Loading and Outflow for detailed description). The latter scenario reduces CO_2_ fixation and amplifies electron transmission to O_2_, resulting in over-yielding of reactive oxygen species (ROS) that injures the photosynthetic apparatus and accelerates leaf chlorosis or necrosis (Cakmak and Kirkby, 2008). Thus, Mg-deficient plants are fairly sensitive to the extent of light intensity. Their leaves swiftly display prominent MGD symptoms when exposed to high light-intensity (Mengutay et al., 2013). Partial shading efficiently suppresses such light-intensity-dependent response, even shading itself does not change Mg concentrations in different leaf parts. Induction of leaf chlorosis by the photooxidative stress under MGD signifies that plants have a higher physiological prerequisite for Mg under high-light intensity conditions (Cakmak and Kirkby, 2008). In addition, lower efficiencies of Mg-chelatase (e.g., low ATP accessibility under MGD) also trigger leaf chlorosis with protoporphyrin IX accumulation in leaves (Cakmak and Kirkby, 2008). MGD may further alter abscisic acid and ethylene signaling, redox status, and metal homeostasis, giving rise to leaf chlorosis and necrosis (Cakmak and Kirkby, 2008).

#### Reduction in the Yield and Quality of Agricultural Commodities

Mg serves as a crucial mineral nutrient for the generation and partitioning of carbohydrates and related biomass accumulation in most crop species (Cakmak and Kirkby, 2008; Chen Z. C. et al., 2018; Koch et al., 2019; Zhang et al., 2020). Sucrose also functions to mediate a wide array of developmental and growth processes as a signaling molecule to fine-tune organ differentiation and genesis for higher crop yield (Hackel et al., 2006; Chen Z. C. et al., 2018). Mg-regulated photosynthesis generates various carbohydrates providing carbon skeletons for the synthesis of a majority of quality components (Gerendás and Führs, 2013). MGD at least disrupts plant photosynthesis and metabolism, and thus affects a range of yield and quality traits of field crops (Gerendás and Führs, 2013; D'Egidio et al., 2019), for instance, cereals (Graeff et al., 2001; Grzebisz, 2013), horticultural crops (Morton et al., 2008; Yang et al., 2012), and forestry (Vacek et al., 2006). Hence, Mg fertilization improves the yield and quality of agricultural produce, and ultimately reduces the risk of rejection when the product is offered for sale (Gerendás and Führs, 2013; Wang et al., 2020; Zhang et al., 2020).

Crops that store a considerable amount of carbohydrates or oil in grain or tuber require adequate Mg supply to optimize their yield and quality traits (Grzebisz, 2013). For instance, sufficient Mg supply is required to ensure optimal yield and quality of wheat (Grzebisz, 2013; Ceylan et al., 2016), rapeseed (Bogdevich and Mishuk, 2006), and apple (von Bennewitz et al., 2011). In particular, Mg application improves dry matter and starch concentrations of potato (Koch et al., 2019), sugar concentrations of sugar beet (Poglodzinski et al., 2021), vitamin C, and protein concentrations in Chinese cabbage (Lu et al., 2021), grain yield and crude protein concentrations in lentil (Azizi et al., 2011), protein and oil contents in soybean (Vrataric et al., 2006), and yield and next-generation seed germination in a wax gourd (Zhang et al., 2020). Mg supply also improves polyphenol, catechin, and amino acid concentrations in black tea (Jayaganesh et al., 2011; Ruan et al., 2012). For grain crops, Mg contents relates to processing traits such as milling performance (Greffeuille et al., 2006; Gerendás and Führs, 2013). However, further field experiments are necessary to corroborate the broad beneficial effects of Mg on crop quality and to deepen our understanding of underlying mechanisms.

Notably, crop plants are exposed to interactive environmental stresses under field-grown conditions, and MGD exaggerates plant vulnerability under these multifactorial stresses and consequently reduces biomass formation and quality traits. Adequate Mg supply can alleviate harmful effects of abiotic stresses through antagonistic competition with cations (i.e., aluminum), enhancing anti-oxidant systems, and modulating protein activities and gene expression (Chen et al., 2012, 2017; Mengutay et al., 2013; Siddiqui et al., 2016; Boaretto et al., 2020; Kibria et al., 2020; Tian et al., 2021). According to a recent meta-analysis, Mg application improves crop yield and agronomic efficiency (AE) by ~8.5% and 34.4 kg kg^−1^, respectively, under diverse growing conditions (Wang et al., 2020). Thus, Mg nutrition definitely calls for more attention to ensure high yield and quality of agricultural commodities toward efficient resource utilization, sustainable agricultural development, and food security although, under certain extreme circumstances, Mg oversupply adversely affects crop development and quality (Kwon et al., 2019).

### Physiological Dissection of MGD

The aforementioned MGD-associated phenotypes are largely attributed to: (i) weakened photosynthetic activities, (ii) impaired source-sink carbohydrate allocation, (iii) reactive oxygen species (ROS) over-yielding and chlorophyll degradation, and (iv) disrupted energy metabolism and protein synthesis.

#### Weakened Photosynthetic Activity

Mg is preferentially required for Chl structuring and functioning to capture solar energy (Verbruggen and Hermans, 2013), with 15–35% of total available Mg in chloroplasts (Cakmak and Yazici, 2010; Guo et al., 2016). Mg insufficiency negatively affects the expression of genes regulating Chl synthesis and functioning such as *ChlG* encoding Chl synthase, *ChlH*, and *ChlI*, respectively encoding Mg-chelatase subunits H and I, *Cab2* encoding Chl a and b binding proteins, and *PPMT* encoding Mg-protoporphyrin IX methyltransferase, which ultimately diminishes photosynthetic efficiencies (Hermans and Verbruggen, 2005; Zhou et al., 2011; Neuhaus et al., 2013). In the chloroplast, Mg ion (Mg^2+^) drapes negative charges on the thylakoid membrane to initiate grana formation (Puthiyaveetil et al., 2017). Rubisco, the most abundant globular protein controlling plant photosynthesis, harvests CO_2_ in the initial step of the Calvin cycle, which requires Mg to bind to the carbamate group and residues Glu194 and Asp193 on the side chain of Rubisco for its activation (Portis, 2003; Hazra et al., 2015). Mg inadequacy reduces net CO_2_ assimilation rates by weakening Rubisco activities (Li J. et al., 2020; Tian et al., 2021); exogenous supplementation of MgSO_4_ can increase Rubisco abundance to optimal levels and improve photosynthesis efficiencies in Mg-deficient plants (Wolf et al., 2019). In C4 and CAM plants, MGD hinders substrate-binding of phosphoenolpyruvate carboxylase and reduces CO_2_ assimilation (Zhao et al., 2012). Other crucial enzymes for starch synthesis, i.e., ADP-glucose pyrophosphorylase also need Mg^2+^ as a cofactor (Stitt and Zeeman, 2012).

In summary, Mg-deficient leaves show sub-optimal photosynthetic CO_2_ assimilation mainly due to less abundant Chl, impaired critical enzymes, and over-accumulation of photosynthates in leaves (Laing et al., 2000; Hariadi and Shabala, 2004; Yang et al., 2019), which is well-exemplified in various plant species such as maize (Jezek et al., 2015), barley (Tränkner et al., 2016; Jaghdani et al., 2020), common bean (Canizella et al., 2015), sugar beet (Hermans et al., 2004), sunflower (Lasa et al., 2000; Tränkner and Jaghdani, 2019), radish (Samborska et al., 2018), spinach (Ze et al., 2009; Jaghdani et al., 2021), fruit trees (Tang et al., 2012; Yang et al., 2012; Peng et al., 2015; Boaretto et al., 2020), and pine seedlings (Laing et al., 2000; Sun et al., 2001) under diverse growth conditions with Mg limitation.

#### Impaired Carbohydrate Loading and Outflow

Phloem loading of carbohydrates involves H^+^-ATPase-mediated establishment of electrochemical gradients and following co-transport of H^+^-sucrose into the phloem. MGD impairs phloem loading by reducing H^+^-ATPase and Mg-ATP activities and the proton gradient, restricting the functioning of sucrose/H^+^ symporters (i.e., *SUT1*) that demand Mg-ATPase for H^+^-motive force, or affecting other yet to be fully understood key processes (Cakmak and Kirkby, 2008; Ayre, 2011). There is a nearly eight-fold reduction in the translocation of 14C-labeled sucrose in sugar beet after 11 days of the MGD treatment (Hermans et al., 2005); resupplying Mg for 12–24 h transiently restores the sucrose transport rate in Mg-deficient plants to the Mg-sufficient level (Cakmak et al., 1994b; Cakmak and Kirkby, 2008). Mg deficient plants over-accumulate sucrose as well as starch in source leaves (Cakmak et al., 1994b; Cakmak and Kirkby, 2008; Gransee and Führs, 2013; Tanoi and Kobayashi, 2015; Koch et al., 2020; Zhang et al., 2020), compared to over-accumulation of starch in N or P deficient leaves and that of sucrose under K deficiency (Cakmak and Kirkby, 2008; Verbruggen and Hermans, 2013; Wakeel and Ishfaq, 2022a). Elevated accumulation of photosynthates in source leaves is observed in plants such as rice (Ding et al., 2006; Cai et al., 2012), *Arabidopsis* (Hermans and Verbruggen, 2005; Hermans et al., 2006), and citrus (Yang et al., 2012) under MGD. This typical observation appears within days, prior to visible leaf chlorosis, for more timely diagnosis in the field (Hermans et al., 2005; Cakmak and Kirkby, 2008; Yang et al., 2019).

#### ROS Scavenging and Chlorophyll Degradation

Mg deficiency hampers photosynthetic electron transport and over-accumulates unutilized energy, and carbohydrate stacking partially decouples light capture and utilization, which together adversely boosts ROS production (Cakmak and Kirkby, 2008; Tanoi and Kobayashi, 2015). ROS scavenging over-consumes ascorbic acid and SH-containing compounds, with involvement of glutathione reductase, superoxide dismutase, ascorbate peroxidase, and catalase, disrupting cellular redox homeostasis, and endangering various physiological processes (Cakmak and Kirkby, 2008; Chou et al., 2011; Yang et al., 2012; Peng et al., 2015). With Mg application, ROS accumulation in Mg-deficient plants declines by 31% compared to control plants (Hauer-Jákli and Tränkner, 2019).

Over-produced ROS in Mg-deficient leaves stimulates expression of *STAY-GREEN (OsSGR)* to trigger Chl degradation (Peng et al., 2019), and sequentially reduces the concentration of Chl b and a (Mittler, 2002; Hermans et al., 2004; Mengutay et al., 2013; Tränkner et al., 2016). Alternatively, over-accumulation of starch in thylakoids of Mg-deficient plants makes chloroplasts rounder and larger (Farhat et al., 2014); more sugar deposition down-regulates the expression of *Cab2* which is involved in encoding Chl pigments (Hermans et al., 2004). ROS also damages the D1 protein in the PSII reaction center and reduces Rubisco abundance in the leaves (Nakano et al., 2006; Zhou et al., 2006; Takahashi and Badger, 2011; Järvi et al., 2013).

ROS generated in the chloroplast thylakoid need to be instantly detoxified before they diffuse to adjacent compartments (Asada, 2006). MGD thus increase antioxidant activities by related enzymes and metabolites in wheat (Mengutay et al., 2013), maize (Tewari et al., 2004), Mulberry (Tewari et al., 2006), and citrus (Tang et al., 2012) in response to ROS oxidation, particularly when exposed to intensive light (Cakmak and Kirkby, 2008). The concentration of antioxidative can be considered as a biochemical indicator to estimate Mg levels in the leaves (Riga et al., 2005).

#### Altered Energy Metabolism and Protein Synthesis

Mg plays a crucial role in energy metabolism. Briefly, light stimulates proton flux into thylakoid space within chloroplasts, which is compensated by Mg^2**+**^ transfer from thylakoid lumen into the stroma (Shaul, 2002). This bi-directional ion movement is required for the establishment of the cross-membrane electrochemical gradient for adenosine triphosphate (ATP) synthesis (photophosphorylation). In ATP, Mg attaches to two negatively charged phosphoryl groups along with two N bases of adenine, thus enabling binding of Mg-ATP to the hydrophobic cleft of the reactive site of ATP demanding enzymes (Marschner, 2012). Nearly 50% of total cellular Mg^2+^ is bound to ATP (Maguire and Cowan, 2002), therefore, Mg^2**+**^ supply directly affects ATP synthesis and provision (Shabala and Hariadi, 2005).

Mg is also required to assemble ribosomal subunits and maintain protein abundance (Yamamoto et al., 2010; Hazra et al., 2015; Chen Z. C. et al., 2018). A single functional ribosomal unit recruits more than 100 Mg^2+^ (Petrov et al., 2012). Mg^2+^ usually attaches with multiple rRNA phosphate groups, equilibrates electrostatic repulsion, and fixates the tertiary structure of 23S rRNA to promote aggregation of ribosomal subunits (Klein et al., 2004). Maize root hairs significantly down-regulate the abundance of most proteins under MGD (Li et al., 2013), indicating that MGD negatively regulates protein synthesis, as confirmed in maize, wheat (Mengutay et al., 2013), and sugar beet (Faust and Schubert, 2016). In leaves, Rubisco accounts for 30% of the total protein (Jensen, 2000) and nearly 40% of stromal proteins (Hazra et al., 2015). Rubisco concentrations decrease under MGD in maize (Jezek et al., 2015), barley (Tränkner et al., 2016), sunflower (Lasa et al., 2000), spinach (Yuguan et al., 2009), citrus (Peng et al., 2015; Li et al., 2017), and *Pinus radiata* (Laing et al., 2000; Sun et al., 2001). Further, proteomic analysis shows considerably reduced activities of Rubisco and its activase (Rca) under MGD in *Citrus sinensis* (Peng et al., 2015).

## Uptake and Transport of Magnesium in Plants

In well-aerated, virtually neutral pH soils, Mg uptake by plant roots largely depends on passive transport along mass flow driven by the transpiration pull and active influx against the electrochemical gradient across the membrane established by pumping protons out of the root cytoplasm (Mayland and Wilkinson, 1989; Marschner, 2012). Hence, the total amount of Mg^2+^ available to plants is primarily determined by Mg^2+^ concentrations in the soil solution, soil pH, the cation exchange capacity of the soil, and the soil water content (Kobayashi et al., 2013; Jezek et al., 2015; Wang et al., 2020). Mg^2+^ has a smaller ionic radius (0.072 nm) and larger hydrated radius (0.428 nm) (Bose et al., 2011), more easily subject to leaching compared to other cations. The uptake rate of Mg^2+^ in a pH 4.5 solution reduces to half compared to in the pH 6.5 solution (Kobayashi et al., 2013). The Mg transporters (MGT/MRS2) *per se* are generic and permit other cations to pass. Competing cations, for instance, K^+^, Ca^2+^, ${\text{NH}}_{4}^{+}$, and/or Na^+^ generally antagonize Mg uptake (Gransee and Führs, 2013; Xie et al., 2021; Garcia et al., 2022; Wakeel and Ishfaq, 2022b).

**Figure 3:**
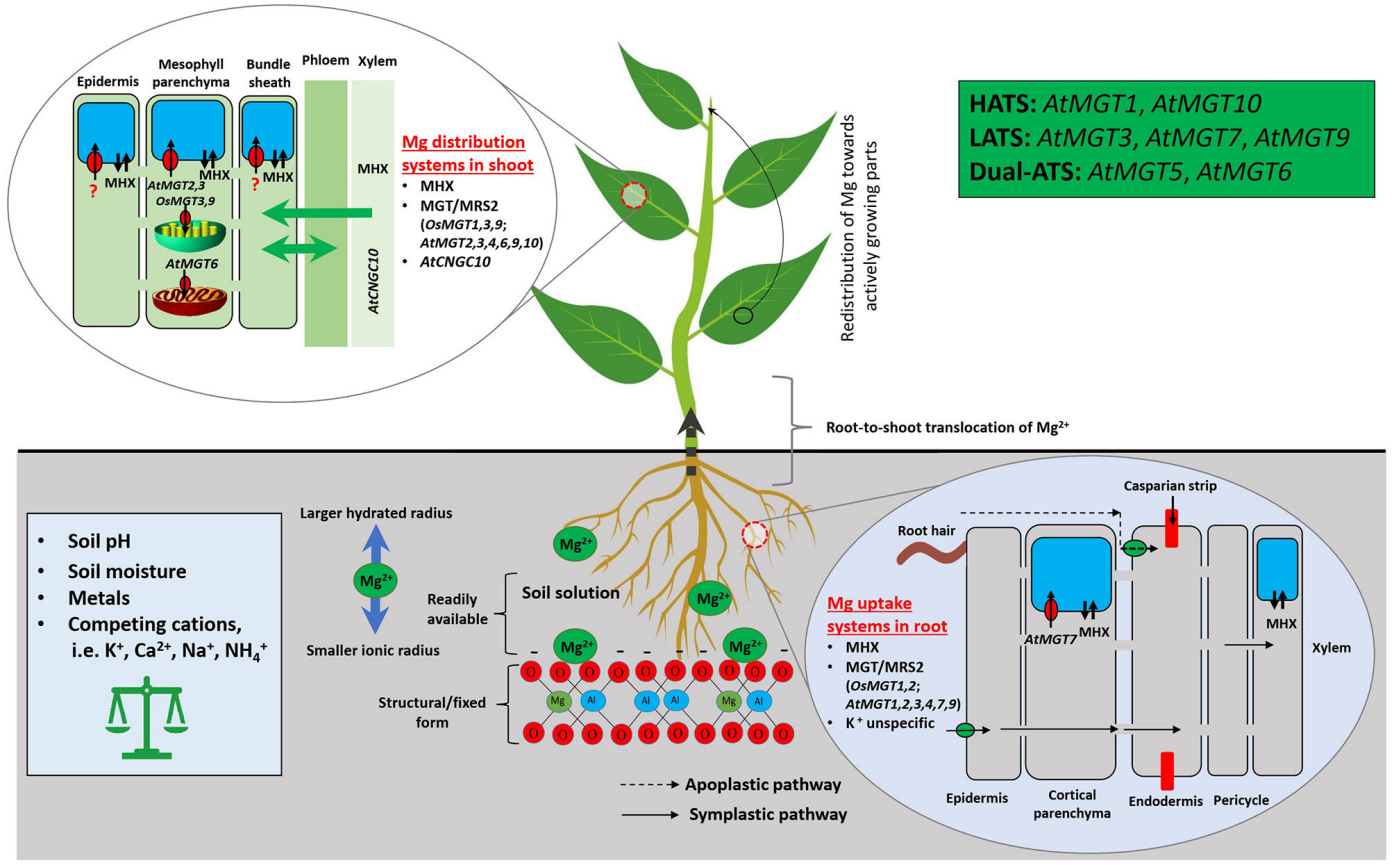
Mg uptake, transport, and homeostasis in model plants *Arabidopsis* and rice. Mg^2+^ absorption from the soil by plants is affected by a range of intrinsic and extrinsic factors. In soil, Mg^2+^ is susceptible to leaching due to its larger hydrated and smaller ionic radius. Once the Mg^2+^ reached the root surface, plants acquire it *via* CorA/MRS2/MGT, unspecific K-transporters, and MHX; using such transporters/exchanger, Mg^2+^ enters into root epidermis, cortex, and then to the xylem after passing through the Casparian strip. The symplastic route depicts Mg movement from cell to cell, whereas, apoplastic refers to movement through extracellular spaces. Followed by, it transfers to shoot and leaves through xylem and stores in vacuole *via* specific transporters/exchanger. MGT/MRS2, Magnesium Transporters; MHX, Mg^2+^/H^+^ Exchanger; MRS, Mitochondrial RNA Splicing; HATS, High-Affinity Transport System; LATS, Low-Affinity Transport System; Dual-ATS, Dual-Affinity Transport System.

A second-level modulator of Mg availability to plants is Mg transporters. CorA, identified during the screening for cobalt (Co) resistant bacterium strains, is the initial Mg transporter mediating the influx of Mg, Co, and nickel into *S. typhimurium* (Hmiel et al., 1986). CorA functionally substitutes for mitochondrial RNA splicing 2 (MRS2) in regulating Mg transport and homeostasis in yeast (Bui et al., 1999; Gregan et al., 2001). Each CorA monomer has two transmembrane helices that only permit divalent cations to move through (Lunin et al., 2006). Homologous to *CorA, MRS2*, and *MGT* encode a 10-member Mg transporter family (referred as the MGT/MRS2 family hereinafter) in *Arabidopsis* (Schock et al., 2000; Li et al., 2001), after pioneering work in functional characterization of the Mg^2+^/H^+^ exchanger (MHX) in Mg transport in higher plant *Arabidopsis* (Shaul et al., 1999). Phylogenetic analysis suggested that MGT/MRS2 family constitutes a three-clade tree *in planta* with two rounds of asymmetric duplications (Ishfaq et al., 2021). The MGT/MRS2 family members function in plants as Mg translocators for root absorption, root-shoot distribution, organelle allocation, and homeostasis (Li et al., 2001, 2016; Marschner, 2012; Saito et al., 2013; Ishfaq et al., 2021). Mg transporters/exchangers in model plants *Arabidopsis* and rice are depicted in Figure 3.

Six *MGT*/*MRS2* members (*MGT1/MRS2-10, MGT2/MRS2-1, MGT3/MRS2-5, MGT4*/*MRS2-3, MGT7/MRS2-7*, and *MGT9/MRS2-2*) may be involved in root uptake and within-plant translocation of Mg^2+^ according to their varying expression patterns in *Arabidopsis* (Gebert et al., 2009). *MGT1*/*MRS2-10* overexpression enhances *Nicotiana benthamiana* tolerance to MGD (Deng et al., 2006), and knockout of *MGT1/MRS2-10* and *MGT2/MRS2-1* makes *Arabidopsis* plants more sensitive to Mg limitation (Lenz et al., 2013). Tonoplast-localized *MGT2/MRS2-1* and *MGT3/MRS2-5* control Mg^2+^ transport into mesophyll vacuoles in *Arabidopsis* (Conn et al., 2011) for osmotic potential adjustment (up to 80 mM Mg^2+^ stored in vacuoles) (Shaul, 2002; Conn et al., 2011). *MGT3/MRS2-5* also partially modulates diel Mg^2+^ fluctuations for optimal Rubisco and photosynthesis activities in rice (Li J. et al., 2020). *AtMGT6/AtMRS2-4*, a dual-affinity transporter, regulates Mg uptake by roots under Mg limitation and plays an essential role in maintaining cellular homeostasis of Mg^2+^ (Mao et al., 2014; Oda et al., 2016; Yan et al., 2018). Mutation of *MGT7/MRS2-7* severely perturbs *Arabidopsis* growth under low Mg (Gebert et al., 2009). Functions of another chloroplast Mg transporter *MGT9*/*MRS2-2* remain to be identified (Saito et al., 2013). In addition, *AtCNGC10* participates in long-distance transportation of Mg^2+^ (Guo K. M. et al., 2010). Further efforts are required to better understand the diverse functions of MGT/MRS2 transporters and underlying regulatory mechanisms for Mg absorption and allocation in plants.

## Spatial Distribution and Widespread MGD in the Farming System of China

As noted in the preceding part, Mg limitation disrupts many fundamental physiological and biochemical processes in plants. Efficient MGD remedy roadmaps for optimum crop production depend on a clear understanding of the distribution pattern and magnitude of MGD in different cultivated regions of the world. For instance, South China has large areas of acidic soils with greater MGD risks due to low pH and intense leaching. Yield-oriented agricultural production maintains a high level of Mg removal from the soil without proper Mg replenishment. With further crop yield increase (projected to ~50% in 2030) (Zhang et al., 2011; Cui et al., 2014) to feed a 1.4 billion population, MGD is becoming a serious limiting factor in crop production in China, which calls for a better understanding of Ex-Mg concentrations in the soil and Mg fertilization strategies across diverse soil, crop, and ecological zones. Here, we provide a focused overview of MGD in China according to the available data to guide balanced fertilization and close yield gaps among different cultivated regions of the country.

The data used in this part of the review regarding soil Ex-Mg (0–20 cm depth, NH_4_OAc-extractable Mg) in distinct croplands of China were extracted from available published articles (field experiments) in English as well as Chinese journals. Soil Ex-Mg in different provinces was also obtained from the website of “National Earth System Science Data Center, National Science and Technology Infrastructure of China” (http://www.geodata.cn), and the recent annual report of “International Magnesium Institute (IMI)” China (http://www.magnesiuminstitute.org/) (details in Supplementary Files 1, 2). The soil database (*n* = 2,544) was compiled from five distinct regions covering 24 provinces (provincial-level administrative divisions) in China. The information about soil pH and climate of different provinces was adapted from previous reports (Han et al., 2011; Li et al., 2015; Chen S. et al., 2018). The country-wise database concerning soil Ex-Mg was assembled from the Food and Agricultural Organization (FAO) of the United Nations (http://www.fao.org/3/a-at167e.pdf).

### Geographical Distribution of Mg in Croplands

**Figure 4:**
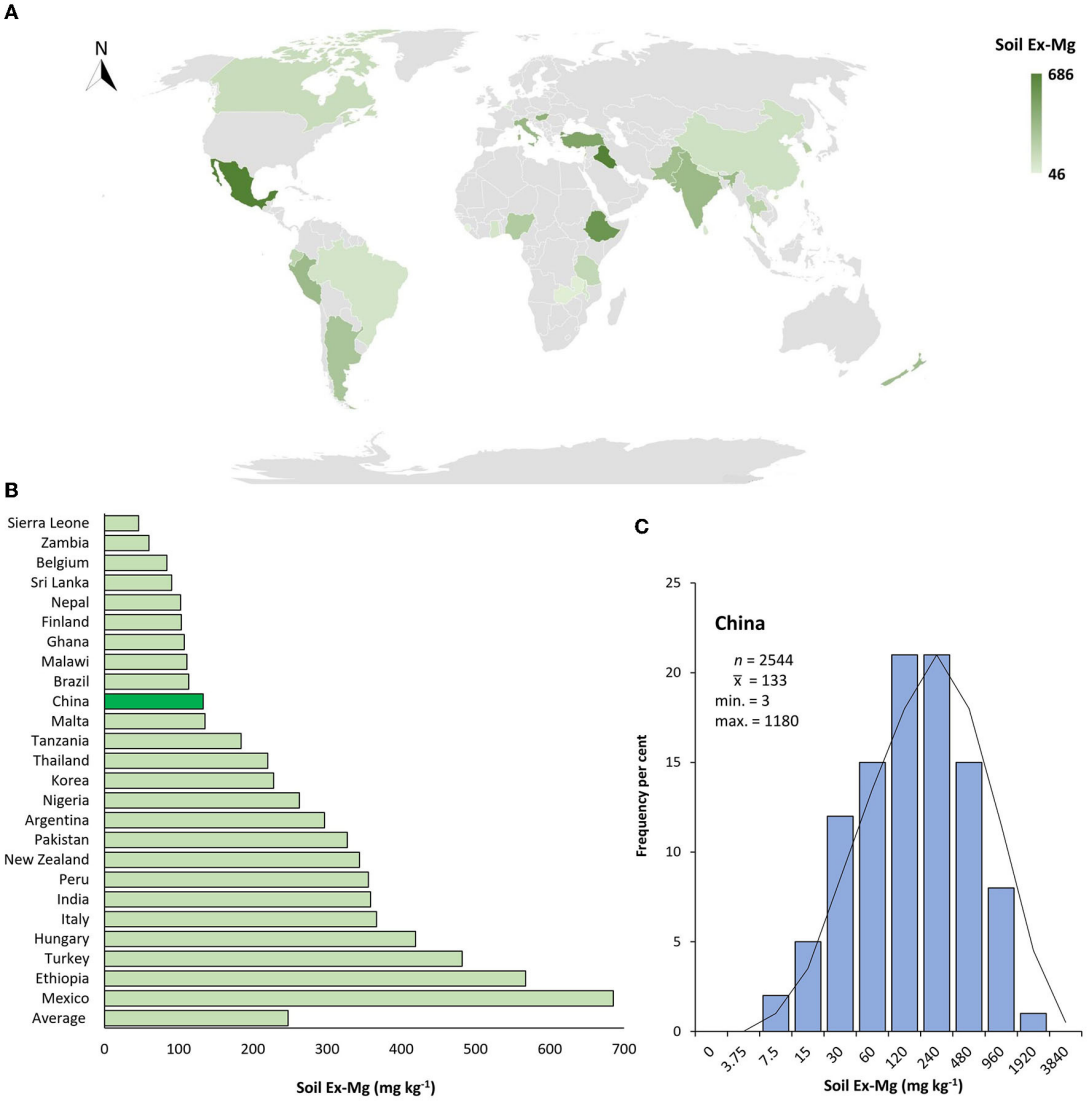
Geographical distribution of soil Ex-Mg in agricultural systems. **(A)** Global soil Ex-Mg status for data available countries, **(B)** Global ranking of soil Ex-Mg for data available countries (value for China is highlighted by the dark green bar), **(C)** Frequency per cent of soil Ex-Mg in China's farming systems. Gray color in map indicated data not found.

Global Ex-Mg distribution in croplands varies dramatically depending on soil and climate types and land utilization practices. With regard to the overall abundance of soil Ex-Mg, China ranked 16th among 25 data-available countries. Many countries such as Mexico, Ethiopia, Italy, India, New Zealand, Pakistan, and Thailand had higher Ex-Mg concentrations in the soil in spite of some other less abundant countries, i.e., Brazil, Malawi, Finland, and Sri Lanka as compared to China (Figures 4A,B).

**Figure 5:**
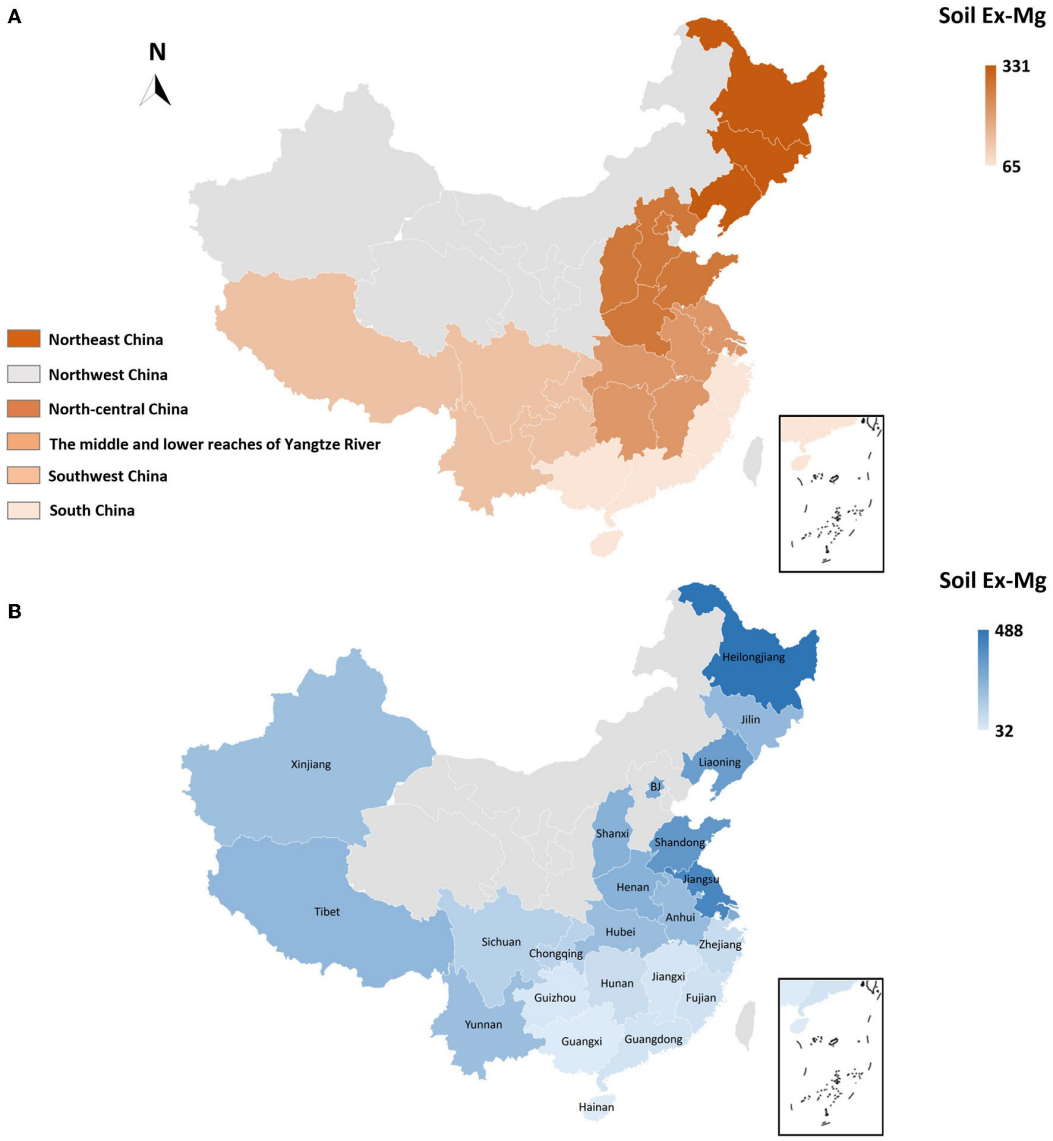
Spatial distribution and magnitude of Mg deficiency in China's farming system. **(A)** Spatial distribution of soil Ex-Mg in distinct regions of China, **(B)** Provincial-level soil Ex-Mg distribution in agricultural soils of China. In China, ~55% of arable lands are Mg-deficient (Ex-Mg < 120 mg kg^−1^), with an obvious spatially declining trend from northern (331 mg kg^−1^ on average) toward southern (65 mg kg^−1^ on average) China. Gray color in maps indicated data not found.

To better interpret the collected data, soil Ex-Mg is categorized into four groups as severely deficient (<30 mg kg^−1^), deficient (31–60 mg kg^−1^), moderate (61–120 mg kg^−1^), and relatively sufficient (>120 mg kg^−1^). Our categorization is well in agreement with previous rating standards (Metson and Brooks, 1975; Metson and Gibson, 1977). Surprisingly, ~55% of croplands in China were Mg-deficient including 7% severely deficient, ~27% deficient, and 21% moderate, and the remaining was Mg sufficient for plant growth (Figure 4C). On the broader perspective, concentrations of soil Ex-Mg generally decreased from northern China to southern China (Figures 5A,B). The mean soil Ex-Mg in China is **~**133 mg kg^−1^ with a range of 227–488 mg kg^−1^ (331 mg kg^−1^ on average) in Northeast China, 251–357 mg kg^−1^ (275 mg kg^−1^ on average) in the north-central region, 54–418 mg kg^−1^ (214 mg kg^−1^ on average) in the middle and lower reaches of the Yangtze River, 49–233 mg kg^−1^ (133 mg kg^−1^ on average) southwest, and 32–89 mg kg^−1^ soil (65 mg kg^−1^ on average) in South China (Figure 5A, Supplementary File 1).

At the provincial-level, Hainan, Guangxi, Guangdong, Fujian, and Zhejiang in South China, Hunan and Jiangxi in the middle and lower reaches of the Yangtze River, and Guizhou in the southwest were found deficient in soil Ex-Mg. In particular, Hainan and Guangxi showed the lowest values 32 and 35 mg kg^−1^, respectively. Chongqing (~127 mg kg^−1^) and Sichuan (~133 mg kg^−1^) approached to the sufficient level, while Shanghai, Jiangsu, Anhui, Shandong, Henan, Shanxi, Beijing, Liaoning, Jilin, and Heilongjiang were categorized sufficient in soil Ex-Mg. The highest provincial concentration was reported as 488 mg kg^−1^ in Heilongjiang. Soil Ex-Mg data were not well-reported in northwestern areas such as Inner Mongolia, Shaanxi, Gansu, Qinghai, and Xinjiang (Figure 5B, Supplementary File 1).

**Figure 6:**
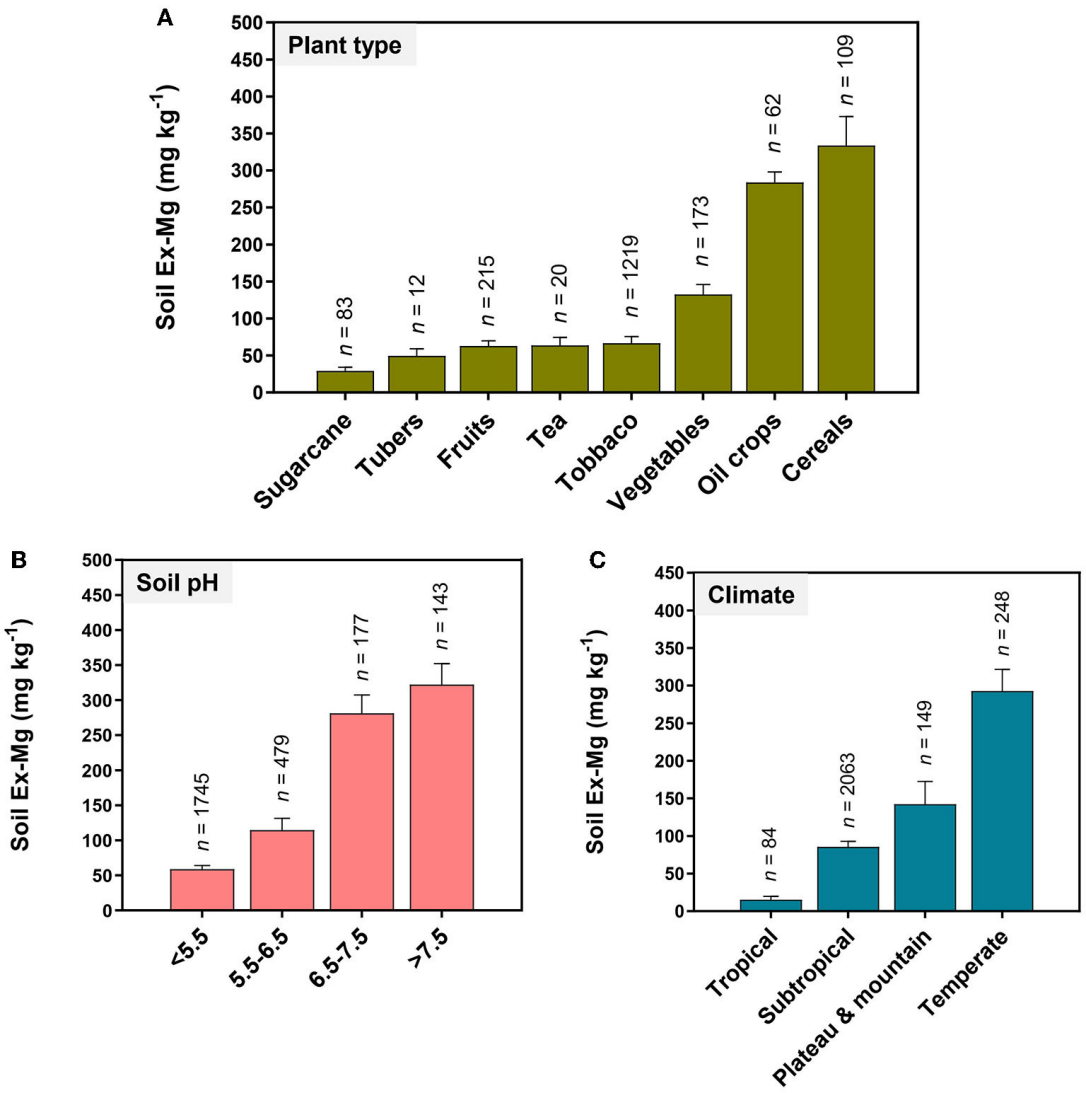
Soil Ex-Mg variations with plant type, soil pH, and climate in China's agricultural system. **(A)** Fruits, vegetables, sugarcane, tubers, tea, and tobacco contribute higher in the depletion of soil Ex-Mg. Mg deficiency becomes increasingly severe **(B)** in acidic soils, and **(C)** in tropical and subtropical climate zones of China.

MGD primarily traced back to Ex-Mg depletion in the soil. Long-term soil mining as a result of intensive cropping accelerates Mg removal. For instance, intensive cultivation of fruits, vegetables, sugarcane, tubers, tea, and tobacco largely contributed to the depletion of soil Ex-Mg (Figure 6A). The amount of Ex-Mg released from parental minerals likely becomes insufficient to support optimum crop production, especially in sandy soils. Chinese farmers usually do not apply Mg fertilizers to replenish the soil, mostly relying on N, P, and K fertilizers (Liu et al., 2003; Zhang and Zhang, 2008; Guo J. H. et al., 2010; Wang et al., 2022). The data presented in Figure 6B shows that MGD becomes increasingly severe in acidic soils and each unit decrease in soil pH from neutral reduced ~two-fold soil Ex-Mg. In South China, acidic soils promoted Mg^2+^ solubilization, and dissolved Mg^2+^ is frequently subject to strong leaching associated with precipitation and irrigation (Guo J. H. et al., 2010; Zhu et al., 2018; Zhang et al., 2021). N over-application further lowers soil pH, exaggerating the MGD situation in certain areas. Climatic conditions also affect Mg dynamics in the soil–plant system. The availability of soil Ex-Mg in different climatic zones of China is found in the following order: temperate > plateau and mountain > subtropical > tropical (Figure 6C). Finally, abundant competing cations such as high concentrations of Ca^2+^ in calcareous soils, ${\text{NH}}_{4}^{+}$, H^+^, and Al^3+^ in acidic soils, or Na^+^ in saline soils, counteracted Mg^2+^ to reduce its availability to crops (Mengel et al., 2001; Gransee and Führs, 2013).

### Responses of Crop Plants to Mg Fertilization

With continuous Mg removal by high-yielding fertilizer-responsive crop varieties without Mg supplementation, MGD is becoming a rising concern for optimum crop production in different cultivated regions of the world (Guo et al., 2016; Senbayram et al., 2016; Wang et al., 2020). Beyond the soil data indicating apparent MGD in China, increasingly more evidence corroborates the responsiveness of different crop plants to Mg supplementation under field conditions. In South China, with frequently occurring severe Mg deficiency, the combined application of Mg and lime in Pinghe, Fujian province, a major pomelo production county, increases yield and total soluble solids by 30.5 and 4.2%, respectively (Zhang et al., 2021). In the same region, Mg application also improves the number of amino acids and total sugars in tea plants in Zhejiang (Ruan et al., 2012) and the yield of wax gourd in Guangzhou under field conditions (Zhang et al., 2020). Field studies in the lower and middle reaches of the Yangtze River, an Mg-deficient region, confirm that Mg fertilization improves the yield, oil content, unsaturated fatty acids of the important regional crop rapeseed (Geng et al., 2021). Mg fertilization also promotes tobacco yield and quality in South Anhui (Zhang et al., 2015). Even in Mg-sufficient soils (northeast and north-central China), field-based studies show 8.5 and 9.3% yield increases in Mg supplemented rice and cabbage plots, respectively (Ding et al., 2012; Liu et al., 2021). Indeed, most crop plants have highly dynamic Mg demand over the growing season and may show MGD symptoms at certain critical growth stages even in Mg sufficient fields, therefore, Mg application improves crop yield or quality in a species-dependent manner (Wang et al., 2020; Liu et al., 2021). How dramatic spatial variations in the abundance of soil Ex-Mg affect crop yield and quality improvement, and what are crop-, soil-, and region-oriented strategic solutions for fertilizer providers have emerged as two fundamental challenges to be tackled seriously.

**Figure 7:**
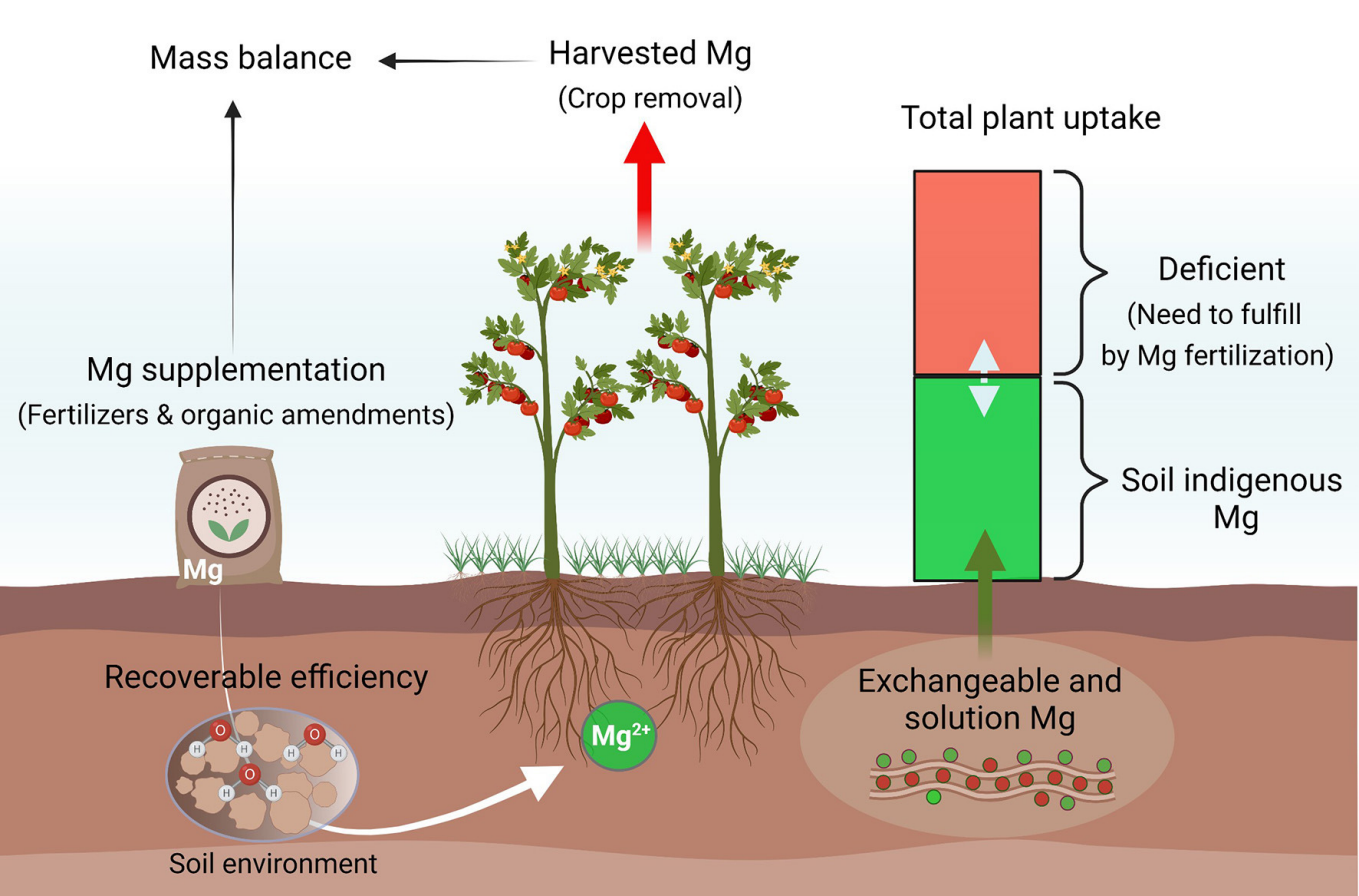
A proposed model to combat rising Mg deficiency in different agroecosystems. Mg availability to plants highly depends on the abundance of indigenous Mg and other soil factors. To close yield gaps in different croplands, Mg recommendations should be optimized primarily according to its net removal and indigenous soil reserves.

Our soil database suggests that agroecosystems in South China, the southwest, and the middle and lower reaches of the Yangtze River are more responsive to Mg fertilization, compared to other cultivated regions in China. These regions are particularly important for the production of cash crops such as vegetables, fruits, tea, and tobacco. Mg fertilization not only prevents crops from potential MGD at the seedling stage but also promotes photosynthesis for higher yield and better quality. Meanwhile, it improves nutrient utilization efficiency by balanced elemental provision and synergistic effects and reduces nutrient loss to the environment (Tian et al., 2021; Wang et al., 2022). Notably, MGD can lead to hypomagnesemia grass tetany (hypomagnesemia), reported in ~30–50% of dairy herds in New Zealand, a foremost reason for the reduction of milk production in grazing animals (Loganathan et al., 2005). Mg supplementation in the grasslands of southwest and northwest China ensures pasture and grazing animal productivity. To efficiently combat rising MGD in agricultural systems, significantly more efforts are required to match soil providing capacities with crop demand by precise plant and soil testing, proper fertilizer formulation, and optimal fertilization rates and timing to minimize net soil mining (Figure 7).

## Concluding Remarks and Way Forward

This review narrates the biological functions of Mg and draws attention to the rising risks of MGD in croplands. Numerous classic literature and the latest explorations agglomerated in this article demonstrate that Mg mediates a wide array of central physiological and biochemical processes in plants, while MGD undoubtedly weakens the performance and productivity of different agricultural systems. In China, ~55% of arable lands are found Mg-deficient (Ex-Mg < 120 mg kg^−1^ soil), with an obvious spatially declining trend of soil Ex-Mg from northern (227–488 mg kg^−1^) toward southern (32–89 mg kg^−1^) China. Mg deficiency can be primarily traced back to higher depletion of soil Ex-Mg by fruits, vegetables, sugarcane, tubers, tea, and tobacco in tropical and subtropical climate zones. Further, each unit decline in soil pH from neutral can reduce ~2-fold Ex-Mg. This focused documentation provides a useful quantitative framework for a wide range of plant scientists and growers to understand Mg dynamics in the soil, and accordingly, to combat the rising MGD for higher crop yield and better quality. It also signifies the call for a global effort to better evaluate the spatial distribution of MGD and related risks.

Although significant advances have been made in understanding the physiological functions of Mg, a large knowledge gap still exists to be closely compared to N, P, and K. Much more efforts are required to investigate molecular and genetic mechanisms of physiological functions of Mg, MGD signaling, and adaptation to local heterogeneous Mg conditions, which lays the foundation for breeding for Mg efficient crop germplasm and cultivars. Genome editing and omics techniques may provide valuable resources for this purpose.

Given spatial variations of soil Ex-Mg, a considerable amount of research work can be projected to study responses of different crops to Mg supplementation and develop site-specific Mg benchmarks in distinct agroecosystems. To counteract competing cations, it is important to rationalize fertilizer formulation and application strategies to improve Mg availability. Finally, creating awareness and eliminating misconceptions among local growers, together with subsidizing Mg fertilizers, especially in Mg-deficient regions, can be beneficial to close yield and quality gaps among different cultivated regions.

## Author Contributions

MI and XL are involved in conceptualization, data curation, analysis, software, writing—original draft, writing—review, and editing. YW, MY, and ZW are involved in data collection. LW and CL are involved in writing—review and editing. All authors revised and approved the submitted version of the manuscript.

## Funding

This work was supported by the National Natural Science Foundation of China (32172663).

## Conflict of Interest

The authors declare that the research was conducted in the absence of any commercial or financial relationships that could be construed as a potential conflict of interest.

## Publisher's Note

All claims expressed in this article are solely those of the authors and do not necessarily represent those of their affiliated organizations, or those of the publisher, the editors and the reviewers. Any product that may be evaluated in this article, or claim that may be made by its manufacturer, is not guaranteed or endorsed by the publisher.
