# Supplementary material for: Physiological Essence of Magnesium in Plants and Its Widespread Deficiency in the Farming System of China
Source: Front Plant Sci. 2022 Apr 25;13:802274. doi: 10.3389/fpls.2022.802274 (PMC9085447; doi:10.3389/fpls.2022.802274)
Supplement: Supplementary file 1 [file Data_Sheet_1.docx]

**Physiological Essence of Magnesium in Plants and Its Widespread Deficiency in the Farming System of China**

Muhammad Ishfaq^1^, Yongqi Wang^1^, Minwen Yan^1^, Zheng Wang^2^, Liangquan Wu^3^, Chunjian Li^1,3^, Xuexian Li^1^*

*^1^College of Resources and Environmental Sciences; National Academy of Agriculture Green Development; Key Laboratory of Plant-Soil Interactions, Ministry of Education, China Agricultural University, 100193 Beijing, China*

*^2^Shaanxi Forestry Bureau, Xi'an, China*

*^3^International Magnesium Institute, Fujian Agriculture and Forestry University, Fuzhou 350002, China*

*** Correspondence:** [steve@cau.edu.cn](mailto:steve@cau.edu.cn)

**Citation:** *Ishfaq M, Wang Y, Yan M, Wang Z, Wu L, Li C and Li X (2022) Physiological Essence of Magnesium in Plants and Its Widespread Deficiency in the Farming System of China. Front. Plant Sci. 13:802274. doi: 10.3389/fpls.2022.802274*

Supplementary Material

**Supplementary Data Sheet S1:** Raw data used for interpretation of soil Ex-Mg concentration in different cropping systems of China

1. Data compiled from English and Chinese journals (a detailed reference file is also attached)

| Province | Crop | Country | Soil Ex-Mg | Unit | Reference |
| --- | --- | --- | --- | --- | --- |
| Guangdong | Banana | China | 134.4 | mg kg^-1^ | Yao et al., 2005 |
| Guangdong | Potato | China | 33.6 | mg kg^-1^ | Huang et al., 2014 |
| Guangdong | Cabbage | China | 49.2 | mg kg^-1^ | Chen et al., 2008 |
| Guangdong | Cabbage | China | 20.4 | mg kg^-1^ | Chen et al., 2008 |
| Guangdong | Cabbage | China | 7.2 | mg kg^-1^ | Chen et al., 2008 |
| Guangdong | Cabbage | China | 18 | mg kg^-1^ | Chen et al., 2008 |
| Guangdong | Cabbage | China | 115.2 | mg kg^-1^ | Chen et al., 2008 |
| Guangdong | Cabbage | China | 181.2 | mg kg^-1^ | Chen et al., 2008 |
| Guangdong | Cabbage | China | 166.8 | mg kg^-1^ | Chen et al., 2008 |
| Guangdong | Cabbage | China | 146.4 | mg kg^-1^ | Chen et al., 2008 |
| Guangdong | Cabbage | China | 154.8 | mg kg^-1^ | Chen et al., 2008 |
| Guangdong | Cabbage | China | 109.2 | mg kg^-1^ | Chen et al., 2008 |
| Guangdong | Cabbage | China | 54 | mg kg^-1^ | Chen et al., 2008 |
| Guangdong | Cabbage | China | 49.2 | mg kg^-1^ | Chen et al., 2008 |
| Guangdong | Cabbage | China | 226.8 | mg kg^-1^ | Chen et al., 2008 |
| Guangdong | Tea | China | 20 | mg kg^-1^ | Ruan et al., 2002 |
| Guangdong | Citrus | China | 18.8 | mg kg^-1^ | Yao et al., 2006 |
| Guangdong | Citrus | China | 49.9 | mg kg^-1^ | Yao et al., 2006 |
| Guangdong | Peanut | China | 75.4 | mg kg^-1^ | Li et al., 2007 |
| Guangdong |  | China | 20 | mg kg^-1^ | Du et al., 1993 |
| Guangdong | Pumelo | China | 49.9 | mg kg^-1^ | Yao et al., 2003 |
| Guangdong | Fruits | China | 179 | mg kg^-1^ | Xu et al., 2000 |
| Guangdong | Citrus | China | 77.9 | mg kg^-1^ | 姚丽贤等, 2006 |
| Guangdong | Vegetables | China | 160 | mg kg^-1^ | 郭巨先等, 2010 |
| Guangdong | Banana | China | 75.4 | mg kg^-1^ | 李国良等, 2007 |
| Guangdong | Tomato | China | 24.6 | mg kg^-1^ | 赵冰等, 2006 |
| Shandong |  | China | 480 | mg kg^-1^ | 杨力等, 1998 |
| Shandong | Tea | China | 91.6 | mg kg^-1^ | 阮建云等, 2002 |
| Shandong |  | China | 320.4 | mg kg^-1^ | 卢丽萍等, 1995 |
| Henan | Tobacco | China | 253.9 | mg kg^-1^ | Liu et al., 1998 |
| Sichuan | Tobacco | China | 70.8 | mg kg^-1^ | Yao et al., 2016 |
| Sichuan | Tobacco | China | 120 | mg kg^-1^ | Yuo et al., 2016 |
| Jiangsu | Potato | China | 276 | mg kg^-1^ | Wen, 2005 |
| Jiangsu | Tea | China | 140.3 | mg kg^-1^ | 阮建云等, 2002 |
| Hebei | Maize | China | 17.62 | mg kg^-1^ | 杨利华等, 2003 |
| Hunan | Tobacco | China | 82.8 | mg kg^-1^ | Fan, 2007 |
| Hunan | Tobacco | China | 61.2 | mg kg^-1^ | Li et al., 2004 |
| Hunan | Tobacco | China | 60 | mg kg^-1^ | Liu et al., 1998 |
| Hunan | Rapeseed | China | 96.5 | mg kg^-1^ | Li et al., 2018 |
| Hunan |  | China | 38.4 | mg kg^-1^ | 张亚莲等, 2008 |
| Hunan | Tobacco | China | 158.8 | mg kg^-1^ | 向鹏华等, 2013 |
| Hunan | Tobacco | China | 177.76 | mg kg^-1^ | 黎娟等, 2012 |
| Hunan | Tobacco | China | 158.04 | mg kg^-1^ | 张国等, 2009 |
| Hunan | Broccoli | China | 204 | mg kg^-1^ | 孙楠等, 2006 |
| Hunan | Tea | China | 46.15 | mg kg^-1^ | 阮建云等, 2002 |
| Hunan | Tobacco | China | 170.4 | mg kg^-1^ | 颜成生, 2006 |
| Hunan | Tea | China | 13.02 | mg kg^-1^ | 朱永兴等, 2003 |
| Anhui | Tobacco | China | 24.03 | mg kg^-1^ | Zhang et al., 2015 |
| Anhui | Rice | China | 284 | mg kg^-1^ | 李孝良等, 2009 |
| Anhui | Tobacco | China | 257.85 | mg kg^-1^ | 王世济等, 2011 |
| Anhui | Tea | China | 33.55 | mg kg^-1^ | 阮建云等, 2002 |
| Anhui | Soybean | China | 253.85 | mg kg^-1^ | 于群英, 2002 |
| Hubei | Tobacco | China | 212.7 | mg kg-1 | 张晓亮等, 2017 |
| Hubei | Tea | China | 208.8 | mg kg^-1^ | 阮建云等, 2002 |
| Hubei |  | China | 169.7 | mg kg^-1^ | 袁家富等, 2002 |
| Zhejiang | Tea | China | 62 | mg kg^-1^ | Ruan et al., 2002 |
| Zhejiang | Tea | China | 69 | mg kg^-1^ | Ruan et al., 2002 |
| Zhejiang | Tea | China | 48 | mg kg^-1^ | Ruan et al., 2002 |
| Zhejiang | Rice | China | 25.75 | mg kg^-1^ | Wang et al., 1999 |
| Zhejiang | Citrus | China | 53.5 | mg kg^-1^ | Qiu, 2015 |
| Zhejiang | Potato | China | 15.1 | mg kg^-1^ | Ning et al., 2005 |
| Zhejiang | Potato | China | 18.5 | mg kg^-1^ | Ning et al., 2005 |
| Zhejiang | Potato | China | 21.3 | mg kg^-1^ | Ning et al., 2005 |
| Zhejiang | Potato | China | 44 | mg kg^-1^ | Ning et al., 2005 |
| Zhejiang | Rice | China | 35.06 | mg kg^-1^ | Liyan et al., 1994 |
| Zhejiang | Rice | China | 15.68 | mg kg^-1^ | 王慧荣等, 2018 |
| Zhejiang | Tea | China | 44.5 | mg kg^-1^ | 阮建云等, 2002 |
| Zhejiang | Tea | China | 41 | mg kg^-1^ | 刘林敏等, 2009 |
| Zhejiang | Tea | China | 28.3 | mg kg^-1^ | 阮建云等, 2002 |
| Zhejiang | Grapefruit | China | 53.3 | mg kg^-1^ | 胡育化, 2013 |
| Guangxi | Tobacco | China | 48 | mg kg^-1^ | Gao et al., 2016 |
| Guangxi | Tobacco | China | 35 | mg kg^-1^ | Gao et al., 2016 |
| Guangxi | Citrus | China | 37.2 | mg kg^-1^ | Wei, 2013 |
| Guangxi | Tomato | China | 196.8 | mg kg^-1^ | Wei et al., 1991 |
| Guangxi | Peanut | China | 166.8 | mg kg^-1^ | He et al., 1992 |
| Guangxi | Peanut | China | 166.8 | mg kg^-1^ | He et al., 1992 |
| Guangxi | Peanut | China | 90 | mg kg^-1^ | He et al., 1992 |
| Guangxi | Peanut | China | 114 | mg kg^-1^ | He et al., 1992 |
| Guangxi |  | China | 76 | mg kg^-1^ | Du et al., 1995 |
| Guangxi | Watermelon | China | 4.37 | mg kg^-1^ | Tan et al., 1995 |
| Guangxi | Watermelon | China | 4.01 | mg kg^-1^ | Tan et al., 1995 |
| Guangxi | Tea | China | 24.2 | mg kg^-1^ | 阮建云等, 2002 |
| Guangxi | Maize | China | 40.4 | mg kg^-1^ | 李伏生, 1997 |
| Yunnan | Tobacco | China | 201.84 | mg kg^-1^ | Lin et al., 2010 |
| Yunnan | Tobacco | China | 202.84 | mg kg^-1^ | Lin et al., 2010 |
| Yunnan | Tobacco | China | 203.84 | mg kg^-1^ | Lin et al., 2010 |
| Yunnan |  | China | 96.42 | mg kg^-1^ | Rui et al., 2004 |
| Yunnan | Tobacco | China | 81 | mg kg^-1^ | Luo et al., 1992 |
| Yunnan | Tobacco | China | 224.5 | mg kg^-1^ | 濮永瑜, 2019 |
| Yunnan |  | China | 523.34 | mg kg^-1^ | 白由路等, 2004 |
| Yunnan |  | China | 565.4 | mg kg^-1^ | 陈建军等, 2012 |
| Yunnan | Tobacco | China | 95 | mg kg^-1^ | 李永忠, 2001 |
| Jiangxi | Tobacco | China | 63 | mg kg^-1^ | Feng et al., 2009 |
| Jiangxi | Citrus | China | 47.17 | mg kg^-1^ | 范玉兰等, 2014 |
| Jiangxi | Citrus | China | 46.81 | mg kg^-1^ | 刁莉华等, 2013 |
| Jiangxi | Tobacco | China | 55.8 | mg kg^-1^ | 冯小虎, 2009 |
| Jiangxi | Tea | China | 46.95 | mg kg^-1^ | 阮建云, 2002 |
| Jiangxi | Citrus | China | 46.81 | mg kg^-1^ | 刁莉华, 2013 |
| Liaoning | Tomato | China | 203.23 | mg kg^-1^ | 刘佳, 2017 |
| Liaoning |  | China | 429 | mg kg^-1^ | 姜勇等, 2003 |
| Fujian | Tea | China | 35 | mg kg^-1^ | Ruan et al., 2002 |
| Fujian |  | China | 37.5 | mg kg^-1^ | Li et al., 2007 |
| Fujian | Banana | China | 28.8 | mg kg^-1^ | Guo, 2005 |
| Fujian | Tea | China | 35 | mg kg^-1^ | Ruan et al., 1997 |
| Fujian | Tea | China | 40.43 | mg kg^-1^ | Lin et al., 2005 |
| Fujian | Watermelon | China | 55.27 | mg kg^-1^ | Lin et al., 2005 |
| Fujian | Cabbage | China | 60.78 | mg kg^-1^ | Lin et al., 2005 |
| Fujian | Citrus | China | 14.4 | mg kg^-1^ | Zhong, 2013 |
| Fujian | Tobacco | China | 45.3 | mg kg^-1^ | Huang et al.,1991 |
| Fujian | Tobacco | China | 14.18 | mg kg^-1^ | Xu et al., 2011 |
| Fujian | Tobacco | China | 17.68 | mg kg^-1^ | Xu et al., 2011 |
| Fujian | Cabbage | China | 37.6 | mg kg^-1^ | Yu, 2015 |
| Fujian | Rice | China | 46.5 | mg kg^-1^ | Yu, 2015 |
| Fujian | Tea | China | 26.72 | mg kg^-1^ | Pan, 2015 |
| Fujian | Rice | China | 16 | mg kg^-1^ | Lin et al., 1990 |
| Fujian | Rice | China | 23 | mg kg^-1^ | Lin et al., 1990 |
| Fujian | Rice | China | 29 | mg kg^-1^ | Lin et al., 1990 |
| Fujian | Rice | China | 44 | mg kg^-1^ | Lin et al., 1990 |
| Fujian | Rice | China | 49 | mg kg^-1^ | Lin et al., 1990 |
| Fujian | Rice | China | 51 | mg kg^-1^ | Lin et al., 1990 |
| Fujian | Tobacco | China | 3.56 | mg kg^-1^ | Hong, 2004 |
| Fujian |  | China | 51.78 | mg kg^-1^ | 林小兰等, 2019 |
| Fujian | Tobacco | China | 34.49 | mg kg^-1^ | 陈星峰, 2005 |
| Fujian | Tea | China | 9.63 | mg kg^-1^ | 侯玲利等, 2009 |
| Fujian | Tobacco | China | 31.78 | mg kg^-1^ | 李春英等, 2000 |
| Fujian | Citrus | China | 57 | mg kg^-1^ | 陈欢欢等, 2019 |
| Fujian | Tea | China | 23.7 | mg kg^-1^ | 穆聪, 2019 |
| Fujian | Banana | China | 73.7 | mg kg^-1^ | 罗光等, 2006 |
| Fujian | Banana | China | 73.2 | mg kg^-1^ | 郭义龙, 2005 |
| Fujian | Tobacco | China | 34.6 | mg kg^-1^ | 张寿南, 2005 |
| Fujian | Tea | China | 25.4 | mg kg^-1^ | 吴洵, 1998 |
| Fujian | Tobacco | China | 37.71 | mg kg^-1^ | 高伟民, 2009 |
| Fujian | Grapefruit | China | 41.42 | mg kg^-1^ | 黄绿林, 2015 |
| Fujian | Grapefruit | China | 56.67 | mg kg^-1^ | 林锋, 2013 |
| Fujian |  | China | 98.4 | mg kg^-1^ | 林齐民等, 1986 |
| Fujian |  | China | 86 | mg kg^-1^ | 陆集卿等, 1985 |
| Heilongjiang | Rice | China | 5.9 | mg kg^-1^ | Wang, 2009 |
| Heilongjiang | Rice | China | 205.6 | mg kg^-1^ | Wang, 2009 |
| Heilongjiang | Rice | China | 168 | mg kg^-1^ | Zhang et al., 2011 |
| Heilongjiang | Rice | China | 428 | mg kg^-1^ | Liu, 2000 |
| Heilongjiang | Maize | China | 624 | mg kg^-1^ | Liu, 2000 |
| Heilongjiang | Wheat | China | 492 | mg kg^-1^ | Liu, 2000 |
| Heilongjiang | Rice | China | 298.54 | mg kg^-1^ | Wang et al., 2013 |
| Heilongjiang | Rice | China | 3.17 | mg kg^-1^ | Gao et al., 2010 |
| Heilongjiang | Rice | China | 282 | mg kg^-1^ | 刘小慧, 2018 |
| Heilongjiang |  | China | 258 | mg kg^-1^ | 刘宏伟, 2000 |
| Heilongjiang | Rice | China | 58.3 | mg kg^-1^ | 李晓鸣, 2002 |
| Shanxi | Potato | China | 115.6 | mg kg^-1^ | Ding et al., 2012 |
| Shanxi | Potato | China | 642 | mg kg^-1^ | Ding et al., 2012 |
| Shanxi | Potato | China | 256 | mg kg^-1^ | Ding et al., 2012 |
| Shanxi | Potato | China | 383.9 | mg kg^-1^ | Ding et al., 2012 |
| Shanxi |  | China | 255.1 | mg kg^-1^ | 丁玉川等, 2012 |
| Shanxi |  | China | 165.21 | mg kg^-1^ | 郭丽娜等, 2008 |
| Shanxi |  | China | 128.22 | mg kg^-1^ | 刘艳等, 2014 |
| Guizhou | Potato | China | 46.9 | mg kg^-1^ | Zhang et al., 2018 |
| Guizhou | Potato | China | 48.6 | mg kg^-1^ | Long et al., 2018 |
| Guizhou | Tea | China | 132.9 | mg kg^-1^ | 阮建云等, 2002 |
| Chongqing | Citrus | China | 224.9 | mg kg^-1^ | 黄翼等, 2013 |
| Chongqing | Tobacco | China | 12.83 | mg kg^-1^ | 徐畅等, 2010 |
| Chongqing |  | China | 133.2 | mg kg^-1^ | 徐畅, 2008 |
| Hainan | Watermelon | China | 12.4 | mg kg^-1^ | Zhang et al., 2010 |
| Hainan | Pepper | China | 6.8 | mg kg^-1^ | Zhang et al., 2011 |
| Hainan | Pepper | China | 14.1 | mg kg^-1^ | Pan et al., 2009 |
| Hainan | Pineapple | China | 10.44 | mg kg^-1^ | 郭继阳等, 2019 |

1. Data obtained from National Earth System Science Data Center, National Science & Technology Infrastructure of China (NESSDC, NSTIC) (<http://www.geodata.cn>)

| No. of Samples | Province | Country | Soil Ex-Mg | Unit | Reference |
| --- | --- | --- | --- | --- | --- |
| 25 | Fujian | China | 64.2 | mg kg^-1^ | NESSDC, NSTIC |
| 58 | Anhui | China | 183.9 | mg kg^-1^ | NESSDC, NSTIC |
| 90 | Jiangxi | China | 60.3 | mg kg^-1^ | NESSDC, NSTIC |
| 87 | Zhejiang | China | 136.41 | mg kg^-1^ | NESSDC, NSTIC |
| 99 | Hunan | China | 88.8 | mg kg^-1^ | NESSDC, NSTIC |
| 34 | Jilin | China | 227.4 | mg kg^-1^ | NESSDC, NSTIC |
| 35 | Liaoning | China | 330.6 | mg kg^-1^ | NESSDC, NSTIC |
| 27 | Guangxi | China | 21 | mg kg^-1^ | NESSDC, NSTIC |
| 23 | Hainan | China | 32.4 | mg kg^-1^ | NESSDC, NSTIC |
| 19 | Shandong | China | 393.6 | mg kg^-1^ | NESSDC, NSTIC |
| 18 | Hubei | China | 153.1 | mg kg^-1^ | NESSDC, NSTIC |
| 10 | Jiangsu | China | 460.8 | mg kg^-1^ | NESSDC, NSTIC |
| 7 | Shanghai | China | 274.8 | mg kg^-1^ | NESSDC, NSTIC |
| 7 | Henan | China | 248.4 | mg kg^-1^ | NESSDC, NSTIC |
| 11 | Guangdong | China | 84.6 | mg kg^-1^ | NESSDC, NSTIC |
| 5 | Guizhou | China | 21 | mg kg^-1^ | NESSDC, NSTIC |
| 7 | Heilongjiang | China | 828 | mg kg^-1^ | NESSDC, NSTIC |
| 6 | Xinjiang | China | 198 | mg kg^-1^ | NESSDC, NSTIC |
| 5 | Tibet | China | 232.6 | mg kg^-1^ | NESSDC, NSTIC |
| 2 | Qinghai | China | 982.5 | mg kg^-1^ | NESSDC, NSTIC |
| 2 | Beijing | China | 294.3 | mg kg^-1^ | NESSDC, NSTIC |

1. Data obtained from International Magnesium Institute (China) annual report-2019 (<http://www.magnesiuminstitute.org/>)

| No. of Samples | Crop | Province | Country | Soil Ex-Mg | Unit | Reference |
| --- | --- | --- | --- | --- | --- | --- |
| 83 | Sugarcane | Guangdong | China | 29.1 | mg kg^-1^ | IMI, 2019 |
| 1199 | Tobacco | Fujian | China | 70.6 | mg kg^-1^ | IMI, 2019 |
| 117 | Pepper | Fujian | China | 105.7 | mg kg^-1^ | IMI, 2019 |
| 57 | Pineapple | Hainan | China | 37.4 | mg kg^-1^ | IMI, 2019 |
| 26 | Pepper | Chongqing | China | 180 | mg kg^-1^ | IMI, 2019 |
| 20 | Pepper | Guizhou | China | 109 | mg kg^-1^ | IMI, 2019 |
| 60 | Rice | Liaoning | China | 538.7 | mg kg^-1^ | IMI, 2019 |
| 60 | Rape | Hubei | China | 313.8 | mg kg^-1^ | IMI, 2019 |
| 41 | Rice | Jiangsu | China | 417.7 | mg kg^-1^ | IMI, 2019 |
| 140 | Orange | Sichuan | China | 146.1 | mg kg^-1^ | IMI, 2019 |
