# Supplementary material for: Physiological Essence of Magnesium in Plants and Its Widespread Deficiency in the Farming System of China
Source: Front Plant Sci. 2022 Apr 25;13:802274. doi: 10.3389/fpls.2022.802274 (PMC9085447; doi:10.3389/fpls.2022.802274)
Supplement: Supplementary file 2 [file Data_Sheet_2.docx]

**Physiological Essence of Magnesium in Plants and Its Widespread Deficiency in the Farming System of China**

Muhammad Ishfaq^1^, Yongqi Wang^1^, Minwen Yan^1^, Zheng Wang^2^, Liangquan Wu^3^, Chunjian Li^1,3^, Xuexian Li^1^*

*^1^College of Resources and Environmental Sciences; National Academy of Agriculture Green Development; Key Laboratory of Plant-Soil Interactions, Ministry of Education, China Agricultural University, 100193 Beijing, China*

*^2^Shaanxi Forestry Bureau, Xi'an, China*

*^3^International Magnesium Institute, Fujian Agriculture and Forestry University, Fuzhou 350002, China*

*** Correspondence:** [steve@cau.edu.cn](mailto:steve@cau.edu.cn)

**Citation:** *Ishfaq M, Wang Y, Yan M, Wang Z, Wu L, Li C and Li X (2022) Physiological Essence of Magnesium in Plants and Its Widespread Deficiency in the Farming System of China. Front. Plant Sci. 13:802274. doi: 10.3389/fpls.2022.802274*

Supplementary Material

**Supplementary File S2:** List of published articles (English as well as Chinese journals) used to extract soil Ex-Mg data in distinct croplands of China

**Shortlisted Articles from English Language Journals**

Chen Qiongxian, Xu Runsheng, Lv Yecheng,Wan Yunqiao, Cao Jian. 2008. Calcium and magnesium nutrients diagnosis index in soil and effect of fertilizing calcium and magnesium on the growth of Chinese cabbage. Chinese Journal of Soil Science.

Ding Yuchuan, Jiao Xiaoyan, Nie Rui, Li lijun,Huang Mingjing. 2012. Effects of combined application of different nitrogen source and magnesium fertilizer on yield, quality and nutrient uptake of potato. Journal of Agriculture.

Du Chenglin, Tan Hongwei, He Tianchun, Huang Hengzhang, Lu Yusi. 1995. Effect of magnesium fertilizer on dryland soil in central Guangxi Soils.

Du Chenlin, Wang Yingming, Wang Huiming, Liang Jianming. 1993. Effect of magnesium fertilizer on some economic crops in magnesium deficient soil. Chinese Journal of Soil Science.

Fan Caiyin. 2007. Study on the yield and qualities formation and growth of flue-cured tobacco under different magnesium fertilizer. Hunan Agricultural University.

Feng Xiaohu, 2009. Study on assessment of soil magnesium condition and magnesium supply technology in tobacco plant area in Jiangxi. Chinese Academy of Agricultural Sciences.

Gao Huajun, Lin Beisen, Wei Zhong, Luo Gang, Hu Yajie. 2016. Effects of magnesium fertilizer application amount on yield, magnesium content and aroma quality of flue-cured tobacco in high calcium soil area. Journal of Shanxi Agricultural Sciences.

Gao Yang, Zheng Guiping, Qian Yongde, Yin Dawei. 2010. Effects of magnesium and potassium application on photosynthetic characteristics and yield of rice. China Rice.

Guo Yilong. 2005. Study on soil magnesium distribution and effect of magnesium application in banana orchard of Zhangzhou. Soil and Fertilizer Sciences in China.

He Tianchun, Tan Hongwei, Dun Chenglin. 1992. Study on the effect of potassium-magnesium fertilizer on the growth of peanut. Soil.

Hong Huoqi. 2004. Effect of different kinds of Mg fertilizer on yield and quality of flue-cured tobacco. Journal of Minxi Vocational and Technical College.

Huang Jichuan, Peng Zhiping, Yu Junhong, Lin Zhijun, Wu Xuena, Yang Linxiang. 2014. Effects of different amount of magnesium application on yield and quality of winter potato. Guangdong Agricultural Sciences.

Huang Yanxiang, Zhong Taoying. 1991. Study on the content of exchangeable magnesium in soil and the effect of magnesium fertilizer in tobacco area. Soil and Fertilizer Sciences in China.

Li Guoliang, Yao lixian, Fu Changying, He Zhaohuang, Tu Shihua. 2007. Study on the effect of combined K -Mg fertilization on banana. Guangdong Agricultural Sciences

Li Mingde, Xiao Hanqian, Yu Chongxiang, He Yinghao, Guo Zhiqiang, Zhang Yiyang, Zhang Haitao. 2004. K and Mg nutrition of soil and fertilization effect on tobacco production in Hunan. Chinese Journal of Soil Science.

Li Xiao-fang, LI Qian, LEI Liqin, TIAN Guisheng, LU Jianwei. 2018. Effects of magnesium application rates on yield and quality of rapeseed under high potassium soil fertility. Hunan agricultural science.

Li Yu, Li Qinghua, He Chunmei, Lin Xinjian. 2007. Effectiveness of combined application for potassium-magnesium fertilizer on crops. Journal of Peanut Science.

Lin Jiang, Zhang Yingcui, Duan Hongwei, Xiayong. 2010. Effects of adding magnesium and boron on yield and quality of flue-cured tobacco in slope land of purple soil. Journal of Yunnan Agricultural University.

Lin Qimin, Lv Bin, Chen Yongliu. 1990. Effect of magnesium fertilizer on rice and the index of magnesium fraction in paddy soil. Journal of Fujian Agricultural College.

Lin Xinjian, Li Yu, Li Qinghua, Wang Fei, He Chunmei. 2005. Effects of applying sulphate -potassium magnesium on yield and quality of pakchoi, tea and watermelon. Soil and Fertilizer Sciences in China.

Liu Guoshun, Fu Yunpeng, Liu Qinghua, Sun Xiaobin, Wan Huixia, Zhang Han. 1998. effects of magnesium level on the growth, yield and quality of flue -cured tobacco. Acta Agriculturae Universitatis Henanensis.

Liu Hongwei. 2000. Magnesium status in black soil around Harbin and its effects on some crops. Northeast Agricultural University.

Liyan, Tang Jianhai, Li Xiufang. 1994. Studies on the effects of magnesium fertilizer on rice and magnesium diagnostic target. Chinese Agricultural Science Bulletin.

LONG Shengbi¬WANG Kun¬HUANG Wanhua¬WU Pingcheng¬ZHANG Yumei. 2018. Effects of Different MgSO_4_ òates on yield and traits of potatoes. Cultivation and cultivation.

Luo Pengtao, Shao Yan. 1992. Effect of magnesium to output qualities and some physiological indices of flued-tobacco. Journal of Yunnan Agricultural University.

National Earth System Science Data Center, National Science & Technology Infrastructure of China” (<http://www.geodata.cn>)

Ning Jianmei, Li Guisong. 2005. Effect of magnesium on yield and economic characters of potato. Rain Fed Crops.

Pan Caizhu. 2015. Effect of boron and magnesium fertilization on tea yield, quality and soil fertility of red-soil tea plantations. Fujian Journal of Agricultural Sciences.

Pan Shunqiu, Zhang Wen, Pan Xiaozhong, Zeng Jianhua, Hu Chunhua. 2009. Study on the application of magnesium sulfate and magnesium fertilizer in capsicum. Mod Agric Sci Technol.

Qiu Chao. 2015. Effects of Ca, Mg and B fertilizers on yield, quality and nutrient accumulation of changshanhuyou fruit. Huazhong Agricultural University.

Ruan Jianyun, Guan Yanliang, Wu Xun. 2002. Status of Mg availability and the effects of Mg application in tea fields of red soil area in China Scientia. Agricultura Sinica.

Ruan Jianyun, Wu Xun,Ha rdter R. 1997. Effects of potassium and magnesium on the yield and quality of oolong tea. Journal of Tea Science.

Tan Hongwei, Zhou Liuqiang, Du Chenlin, He Tianchun. 1995. Effect of watermelon on potassium and magnesium fertilizer. Guangxi Agricultural Sciences.

Wang Andong, Mu Yonghong, Kong Yu, Sui Yanghui, Wang Liping, Sui Guifang. 2013. Effect of magnesium and potassium combined application on rice yield in cold region. China Rice.

Wang Guangqiang, Pan Xiu, Jiang Zhiming, Zhao Lifang. 1999. Study on comprehensive deficiency of single-cropping hybrid rice and its correction techniques. Journal of Zhejiang Agricultural Sciences.

Wang Xinbing. 2009. Effects of silicon potassium and magnesium on rice growth and yield in paddy soil. Heilongjiang Bayi Agricultural University.

Wei Huokai. 2013. Effect of potassium and magnesium fertilizer on yield and quality of sugar citrus. Agricultural Science Technology and Information.

Wei Qiguang, Tao Sheng, Du Chenglin. 1991. Effect of potassium and magnesium on yield and quality of tomato. Chinese Agricultural Science Bulletin.

Wen Zhanghui. 2005. Effect of magnesium fertilizer on potato in northern Jiangsu. Chinese Potato Journal.

Xu Peizhi, Chen Jiansheng, Zhang Fabao, Tang Shuanhu. 2000. Study on the effect of magnesium on banana, litchi and longan. Guangdong Agricultural Sciences.

Xu Qian, Chen Aiguo, Dai Peigang, Zheng Guojian, Chen Zhihou. 2011. effects of rational application of magnesium fertilizers on growth, yield and quality of flue-cured tobacco. Chinese Tobacco Science.

Yao Li, Pang Liangyu, Lin Cahowen, Li Bin, Guo Shiping, Luo Dingqi. 2016. effect of different magnesium application levels on yield and quality of flue-cured tobacco in Luzhou. Journal of Sichuan Agricultural University.

Yao lixian, Li Guoliang, Xu Chaoxuan, Zhou Xiuchong. 2006. Application effect of magnesium fertilizer on litchi and citrus. Phosphate & Compound Fertilizer.

Yao Lixian, Zhou Xiuchong, Hou jianquan, Yang Jing, Cai Yongfa, Chen Wanzhen. 2003. Effect of combination of K and different forms of Mg and S on Shatian pumelo. Soils & Fertilizers.

Yao lixian, Zhou Xiuchong, Peng Zhiping, Chen Wanzhen. 2005. Nutritional characteristics and K and Mg fertilizer combination in Baxi banana. Plant Nutrition and Fertilizer Science.

Yin Rui, Zhang Chendong, Zhang Yuelin, Qu Shengbin, Song Yuchuan, Huo Yuchang, Ya Ping. 2004. Zn, Mg on yield and quality of oriental tobacco. Journal of Yunan Agricultural University.

Yu Guanglan. 2015. Effect of applying agriculture-used magnesium hydroxide on the yield of cabbage, late rice and content of soil magnesium. Chinese Agricultural Science Bulletin.

Yu Wei, Zhang zongjin, Pang Liangyu, Hu Jianxin, Guan Yu, Yao Li. 2016. Effects of magnesium fertilizer application amount on yield and quality of flue-cured tobacco in panzhihua. Chinese Agricultural Science Bulletin.

Zhang Guo, Zhu Qifa, Xiang Zhihua, Zhu Yinghua, Xia Dinggui, Ji Xuejun. 2015. The influence of magnesium fertilizer on flue-cured tobacco growth yield and quality in South Anhui. Soils.

Zhang Liping, Huang Shaofeng, Kong Yu, He Mei, Chen Shaolong, Gao Yang, Du Jinling, Lu Baiqian. 2011. Effects of silicon potassium and magnesium fertilizer on the yield of rice. Chinese Agricultural Science Bulletin.

Zhang Wen, Pan Shunqiu, Fu Chuanliang, Pan Xiaozhong, Zeng Jianhua, Hu Chunhua. 2010. Effect of potassium and magnesium fertilizer on watermelon. Mod Agric Sci Technol.

Zhang Wen, Xie Liangshang, Ji Qingmei, Fu Chuanliang, Pan Shunqiu, Liu Guobiao. 2011. Response of *Capsicum annuum* to nitrogen, phosphorus, potassium and magnesium. Journal of Agricultural.

Zhang Yumei, Long Shengbi, Huang Wanhua, Wu Pingcheng, Wang Kun. 2018. Field experiment of calcium and magnesium fertilizer on winter potato in jinping county. Agro-technical extension.

Zhong Juwen. 2013. Effect of applying dolomite powder on montante navel orange orchard. Fujian Science and Technology of Rice and Wheat.

**Shortlisted Articles from Chinese Language Journals**

白由路,金继运,杨俐苹.我国土壤有效镁含量及分布状况与含镁肥料的应用前景研究[J].土壤肥料, 2004(02): 3-5.

陈建军,沈晗,招启柏,朱卫星,周冀衡.云南腾冲火山区域主要植烟区土壤养分状况分析[J].作物研究, 2012, 26(02):157-160.

陈欢欢,王玉雯,张利军,罗丽娟,叶欣,李延,陈立松,郭九信.我国柑橘镁营养现状及其生理分子研究进展[J].果树学报, 2019, 36(11): 1578-1590.

陈星峰. 福建烟区土壤镁素营养与镁肥施用效应的研究[D].福建农林大学,2005.

丁玉川,焦晓燕,聂督,程滨,赵瑞芬,刘平.山西农田土壤交换性镁含量、分布特征及其与土壤化学性质的关系[J].自然资源学报, 2012, 27(02): 311-321.

刁莉华,彭良志,淳长品,凌丽俐,李勋,薛珺,范玉兰,钟八莲.赣南脐橙园土壤有效镁含量状况研究[J].果树学报, 2013, 30(02): 241-247.

范玉兰,卢映琼,巫辅香,薛珺,李勋,钟八莲.赣南地区脐橙园土壤交换性钙镁含量分布特征研究[J].中国果树, 2014(03): 29-32.

冯小虎. 江西烟区植烟土壤镁素丰缺状况评价及施镁技术研究[D].中国农业科学院, 2009.

郭巨先,陈琼贤,曹健,赫新洲,李强,李妙汉,高惠楠.广东主要菜田土壤养分状况及施肥建议[J].中国蔬菜,2010 (08): 41-45.

胡育化. 连续两年施用钙、镁、硼肥对常山胡柚叶片黄化和果实品质的影响[D].华中农业大学, 2013.

侯玲利,陈磊,郭雅玲,葛洪力,王果,连伟.福建省铁观音茶园土壤镁素状况研究[J].植物营养与肥料学报, 2009,15 (01): 133-138.

黄绿林.平和县琯溪蜜柚果园土壤镁含量与缺镁矫治[J].东南园艺, 2015, 3(05): 5-7.

黄翼,彭良志,凌丽俐,曹立,王男麒,周薇,邢飞.重庆三峡库区柑橘镁营养水平及其影响因子研究[J].果树学报,2013, 30(06): 962-967.

郭义龙.漳州香蕉园土壤镁素分布状况及施镁效应研究[J].土壤肥料, 2005(02): 38-41.

高伟民. 南平烟区镁素营养状况与施镁效应研究[D].湖南农业大学, 2009.

郭丽娜,程滨,赵瑞芬.太原市土壤交换性镁含量及分布特征[J].山西农业科学, 2008(09): 40-42.

郭继阳,张汉卿,杨越,杨劲明,唐浩真,邓燕,阮云泽,赵艳.基于因子-聚类分析的菠萝园土壤养分状况评价[J].土壤通报, 2019, 50(01): 137-143.

姜勇,张玉革,梁文举,闻大中,乔德波.沈阳市郊耕地不同土属交换态钙镁铁锰铜锌含量状况的分析[J].农业系统科学与综合研究, 2003(03): 207-210-213.

李国良,姚丽贤,付长营,何兆桓,涂仕华.香蕉钾镁配施效应研究[J].广东农业科学, 2007(01): 45-47.

卢丽萍,高贤彪,吴建明,高弼模,杨果,管力生,杨光.山东省土壤代换性镁含量分布特征及评价[J].山东农业科学, 1995(06): 28-30.

黎娟,邓小华,周米良,刘逊,田茂成,田峰,冯晓华,吴秋明.湘西植烟土壤交换性镁含量及空间分布研究[J].江西农业大学学报, 2012, 34(02): 232-236.

李孝良,赵利凤,于群英.安徽省水稻土中镁含量及其影响因素[J].安徽科技学院学报, 2009, 23(05): 17-22.

刘林敏,徐火忠,李贵松,宁建美.松阳县茶树缺镁症的发生与防治[J].广东茶业, 2009 (01): 24-25.

李伏生.广西主要母质土壤交换性镁含量和影响玉米对镁吸收的因素[J].广西农业科学,1997 (03): 25-28.

李永忠,丁善荣,罗济,谢勇,罗鹏涛.不同供水状况对烤烟镁元素吸收和分配的影响[J].云南农业大学学报, 2001(01): 42-45.

刘佳. 长期偏施氮肥对菜田土壤钙、镁、硫含量及番茄品质产量的影响[D]. 沈阳农业大学, 2017.

林小兰.寿宁县耕地地力存在的问题与改良措施[J].基层农技推广, 2019, 7(10): 111-112.

李春英,高伟民,陈腊梅,熊德中.福建烟区土壤镁营养状况及其施用效果研究[J].河南农业大学学报, 2000(01): 63-66.

罗光,郑长焰.蕉园土壤镁、硫营养状况及其肥料效应研究[J].龙岩学院学报, 2006(06): 85-87.

林锋. 琯溪蜜柚果园钙、镁、硫营养状况及缺镁矫治措施研究[D].福建农林大学, 2013.

林齐民,陈举鸣.福建省主要土壤类型的镁素含量[J].福建农学院学报, 1986(02): 132-140.

陆集卿,陈大勋,李双霖.福建省主要土类代换性钾、钙、镁含量及其分布规律的研究[J].福建农学院学报, 1985(02): 134-142.

刘小慧. 黑龙江稻田土壤钙镁丰缺状况及施镁效应研究[D].东北农业大学, 2018.

刘宏伟. 哈尔滨地区黑土镁素供应状况及镁肥肥效的研究[D].东北农业大学, 2000.

李晓鸣.矿质镁对水稻产量及品质影响的研究[J].植物营养与肥料学报, 2002(01): 125-126.

刘艳,姚延梼,李彩珍,陈菲儿,许建卿.晋中市左权县作物土壤有效镁营养状况分析[J].天津农业科学, 2014, 20(08): 38-41.

穆聪. 乌龙茶产区氮素供应状况及对茶树镁营养的影响[D].福建农林大学, 2019.

濮永瑜,毛春堂,潘义宏,周芳芳,马旭,陈月舞.保山市植烟土壤交换性镁含量及空间分布[J].贵州农业科学, 2019, 47(03): 50-53.

孙楠,曾希柏,高菊生,王伯仁.含镁复合肥对黄花菜生长及土壤养分含量的影响[J].中国农业科学, 2006(01): 95-101.

王世济,崔权仁,刘小平,赵第锟,武家美.安徽省烟区土壤和烟叶镁含量状况与镁肥应用研究[J].安徽农学通报(上半月刊), 2011, 17(01): 82-83-98.

王慧荣.泰顺县稻田土壤镁含量及水稻施镁的效果[J].浙江农业科学, 2018, 59(02): 222-223.

吴洵.安溪茶园土壤镁含量和施肥建议[J].福建茶叶, 1998(02): 23-26.

向鹏华,单雪华,王国平,郭维.衡阳烟区土壤镁及烟叶镁含量测定[J].湖南农业科学, 2013(13): 53-54-58.

徐畅,高明,谢德体,慈恩.重庆市植烟区土壤镁素含量状况及施镁效应研究[J].植物营养与肥料学报, 2010, 16(02): 449-456.

徐畅. 重庆市植烟区土壤镁素营养及施镁效应研究[D].西南大学, 2008.

阮建云,管彦良,吴洵.茶园土壤镁供应状况及镁肥施用效果研究[J].中国农业科学, 2002(07): 815-820.

颜成生. 衡南植烟土壤肥力及其与烟叶质量的关系[D].湖南农业大学, 2006.

于群英.安徽沿淮地区土壤交换性镁含量及镁对大豆营养的影响[J].安徽农学通报, 2002(06): 60-62.

袁家富,邹焱,彭成林,章新军,王瑞.鄂西南烟区土壤的主要肥力特征分析[J].湖北农业科学, 2002(01): 38-40.

杨力,刘光栋,宋国菡,泉维洁,卢桂菊,丁光国.山东省土壤交换性镁含量及分布[J].山东农业科学,1998 (03): 6-10.

姚丽贤,周修冲,彭智平,李国良.广东省柑桔园土壤养分肥力研究[J].土壤通报, 2006 (01): 41-44.

杨利华,郭丽敏,傅万鑫.玉米施镁对氮磷钾肥料利用率及产量的影响[J].中国生态农业学报, 2003(01) :84-86.

赵冰,毛小云,廖宗文.几种镁肥对番茄肥效的比较研究[J].土壤通报, 2006(04): 830-832.

张国,赵松义,相智华,关广晟,朱列书,朱英华.湖南烤烟烟叶中镁与土壤交换性镁含量的特征及关系分析[J].中国烟草科学, 2009, 30(04): 52-55.

朱永兴,陈福兴.红壤丘陵茶园镁营养调控研究[J].茶叶科学, 2003(S1): 34-37.

张晓亮,刘丹,杨继龙,柴利广,张凯,王中云.秦巴山烟区(十堰)植烟土壤养分状况分析[J].安徽农业科学, 2017, 45(27): 137-141.

张寿南.闽西北山区烟—稻轮作制中烤烟镁营养问题及施镁效果[J].土壤肥料, 2005(02): 55-57.
